# Effect of Home-based Telerehabilitation on Balance, Functional Mobility, and Quality of Life in Persons with Parkinson’s Disease: A Systematic Review and Meta-Analysis

**DOI:** 10.63144/ijt.2025.6725

**Published:** 2025-12-12

**Authors:** Arnold Fredrick D’Souza, Akhila Jagadish, Jennifer V. D’Souza, Dorcas B.C. Gandhi, Dushyanth Babu Jasti, Manikandan Natarajan

**Affiliations:** 1 Department of Physiotherapy, Manipal College of Health Professions, Manipal Academy of Higher Education, Manipal, Karnataka, India; 2 Symbiosis College of Physiotherapy, Symbiosis International (Deemed University), Pune, Maharashtra, India; 3 Providence Health Care, St. Paul’s Hospital, Vancouver, British Columbia, Canada; 4 College of Physiotherapy and Department of Neurology, Christian Medical College and Hospital Ludhiana, Ludhiana, Punjab, India; 5 Department of Neurology, STAR Hospitals, Banjara Hills, Hyderabad, Telangana, India

**Keywords:** Continuity of care, Digital health, Remote monitoring, Resource limited settings, Telerehabilitation

## Abstract

**Background:**

Persons with Parkinson’s disease (PwPD) require ongoing rehabilitation to maintain independence, but traditional center-based and unsupervised home programs have limitations in accessibility and adherence. Home-based telerehabilitation (TR) offers a promising alternative by enabling remote delivery of exercise interventions.

**Objective:**

To evaluate the effect of home-based TR on balance, functional mobility, and quality of life in PwPD.

**Methods:**

A comprehensive electronic search was conducted across PubMed, CINAHL, Embase, OvidSP, ProQuest, Scopus, Web of Science, Cochrane CENTRAL, and PEDro databases. Interventional studies on exercise-centric home-based TR for PwPD with either balance, functional mobility, or quality of life as outcomes were included.

**Results:**

A total of 37 studies were included in this systematic review, of which 13 were eligible for meta-analysis. The meta-analysis revealed small but significant improvements in balance (SMD = 0.25; 95% CI: 0.04 to 0.45; p = 0.02). and functional mobility (SMD = −0.28; 95% CI: −0.52 to −0.05; p = 0.02). However, no significant effect was observed for quality of life (SMD = −0.08; 95% CI: −0.25 to 0.09; p = 0.35).

**Conclusion:**

Home-based TR is effective for improving balance and functional mobility in PwPD, although, its effect on quality of life is unclear which warrants further research.

There is a rise in the global burden of Parkinson’s disease (PD) (Ferrari et al., 2024; Su et al., 2025). PD is progressive and often leads to postural instability and gait disorder (PIGD) as the condition advances. It has been found that persons with PD (PwPD) who develop PIGD exhibit an accelerated disease progression and considerable deterioration of both motor and cognitive abilities (Jankovic et al., 1990; Thenganatt & Jankovic, 2014; van der Heeden et al., 2016). The falls which result from PIGD in PwPD leads to significant decline in their quality of life and has been associated with increased risk of institutionalization and higher mortality rate (Aamodt et al., 2023; Factor et al., 2011). Thus, it is imperative to preserve the functional independence of PwPD through the timely and effective management of PIGD-related symptoms.

PD is typically managed using an interprofessional approach which incorporates medical, surgical, and exercise-based interventions (Weise et al., 2024). Despite dopaminergic pharmacotherapy being commonplace for the treatment of PD, it has limited efficacy in management of PIGD specifically (Smulders et al., 2016; Tarazi et al., 2014; Vu et al., 2012). Furthermore, it has been found that these medications could paradoxically lead to worsening of gait and result in on-phase freezing in those with advanced PD (Chen, 2012). Despite the efficacy of surgical approaches such as deep brain stimulation in the management of motor symptoms of PD, the high cost of such interventions keeps it out of reach for most individuals, which limits its widespread utility (Pourahmad et al., 2023).

In contrast to medical and surgical interventions, exercise therapy appears to be a cost-effective strategy which has demonstrated short-term improvement in balance and functional mobility resulting from PIGD (Bhalsing et al., 2018). The effectiveness of endurance training, strength training, and physical activity promotion has been highlighted in literature (Ernst & Kalbe, 2023; Schootemeijer et al., 2020b; Zhou et al., 2022). This potentially suggests them as essential components of exercise therapy for PD. It has been reported that regular home-based exercise can enhance physical activity levels and lead to improvement in quality of life in PwPD (Yang et al., 2023). For optimal outcome, early adoption of such interventions is recommended (Ellis et al., 2021).

For the sustenance of functional independence in PwPD, long-term management is essential. An individually tailored home exercise program (HEP) is the cornerstone of long-term physiotherapy management of progressive conditions such as PD. The effects of home-based exercise interventions have been studied extensively in PwPD. Studies have reported it to be effective in enhancing balance and functional mobility in PwPD (Flynn et al., 2019; Yang et al., 2023). It is notable that the outcomes between home-based and center-based exercise were comparable. Additionally, HEP empowered PwPD to sustain regular physical activity levels for an extended duration within limited resources (Flynn et al., 2019). Nevertheless, due to limited adherence posing a significant challenge dampens the effectiveness of HEP. Myriad factors such as low motivation and lack of supervision could be responsible for the lower adherence to HEP among PwPD (Ellis et al., 2013; Kim et al., 2025; Schootemeijer et al., 2020a; Wang et al., 2023). Although supervised in-person center-based physiotherapy interventions could improve engagement, they have limited utility in long-term rehabilitation due to geographical and economic barriers that might prevent the PwPD from visiting the center frequently enough to improve outcomes (Velez et al., 2023).

There has been a significant surge in research and clinical interest in remote healthcare delivery models since the COVID-19 pandemic. Telerehabilitation (TR), is a subset of telemedicine, and is defined as “the use of information and communication technology to provide rehabilitation services to people remotely in their homes or other environments” (Brennan et al., 2009). TR is commonly administered through two delivery methods: synchronous and asynchronous. Synchronous TR involves real-time interaction with patients using videoconference or telephone calls, while asynchronous TR usually includes the sharing of documents, images, or videos through email or mobile messaging platforms for future use (Pramuka & van Roosmalen, 2009).

TR appears to be a cost-effective intervention which could improve accessibility of rehabilitation interventions to a wider population, especially those in lower resource settings with a lack of transportation. Such interventions seem to be especially important for PwPD due to their lower functional mobility and independence which pose as a significant challenge for frequently visiting the center for conventional in-person rehabilitation services. TR allows for the remote administration and supervision of HEP which could allow PwPD to safely engage in exercise at home. Additionally, this could boost the adherence to HEP among PwPD. In summary, it appears that home-based TR is a convenient, economical, and personalized approach to the management of PD (Grigorovich et al., 2022; Vellata et al., 2021).

An earlier review published in 2021 assessed the effect of TR in motor and non-motor symptoms of PD which included balance, gait, quality of life, and adherence (Vellata et al., 2021). After the COVID-19 pandemic, there was an acceleration in the rate of adoption of TR, seen as a surge of TR research since that review. While the current review is focused exclusively on studies employing home-based TR, the search strategy is comparatively more comprehensive via the inclusion of a greater number of databases, as well as the employment of meta-analysis and study-quality assessment.

The aim of the current systematic review is to determine the current evidence on the effect of home-based TR on balance, functional mobility, and quality of life in PwPD in comparison to standard rehabilitation delivered either at home or at the center. Additionally, this review will investigate the factors which influence the outcome of home-based TR in PwPD.

## Methods

This systematic review has been reported in accordance with the Preferred Reporting Items for Systematic Reviews and Meta-Analyses (PRISMA) 2020 guidelines (Page et al., 2021). The review protocol was prospectively registered with PROSPERO (CRD42022339697).

### Search Strategy

An electronic search was conducted across multiple databases, including PubMed, CINAHL (Cumulative Index to Nursing and Allied Health Literature), Embase, OvidSP, ProQuest, Scopus, Web of Science, Cochrane CENTRAL, and the Physiotherapy Evidence Database (PEDro), from inception through May 2025. Controlled vocabulary specific to each database was utilized, along with relevant keywords such as ‘Parkinson’s disease’, ‘Telemedicine’, ‘Telerehabilitation’, ‘Postural balance’, ‘Mobility’, and ‘Quality of life,’ including their synonyms. Boolean operators ‘AND’ and ‘OR’ were used to combine search terms appropriately. A detailed search strategy for all the databases searched is shown in Appendix A.

### Eligibility Criteria

Inclusion criteria encompassed interventional studies involving adults diagnosed with idiopathic PD who received remotely delivered exercise therapy, with outcomes related to balance, functional mobility, or quality of life. Comparators could be a standard in-person center or home-based rehabilitation, unsupervised home exercise, or standard care without any exercise-based intervention. Eligible study designs included randomized controlled trials (RCTs), quasi-experimental studies, single-group pre-post studies, and case reports or series. Observational studies were excluded. Studies were also excluded if the intervention was not delivered in a home setting. Additional exclusions included conference abstracts, posters, and dissertations.

### Study Selection

The Rayyan web application was used to identify and remove duplicate records, with two reviewers (AFD and AK) independently verifying duplicates before deletion to minimize errors. Both reviewers also independently screened titles, abstracts, and full texts to assess study eligibility. Any disagreements were resolved through discussion with a third reviewer (MN).

**Figure 1:**
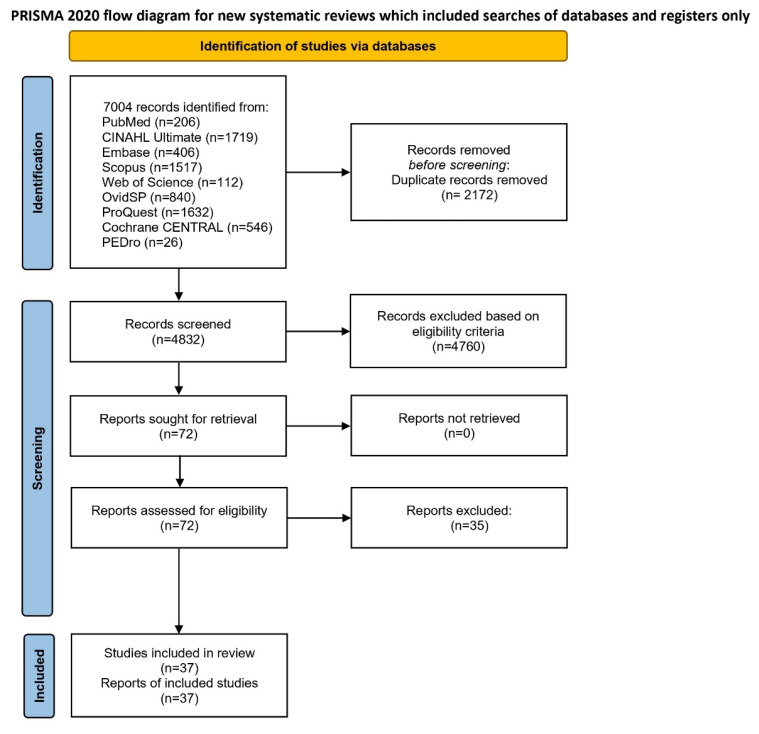
PRISMA Flow Diagram

Figure 1 shows the PRISMA flow diagram detailing the stepwise process of study selection. Comprehensive searches across all the selected databases resulted in the identification of 7004 records. After removing 2172 duplicates via the Zotero software application (version 7.0.2), 4832 records remained for title and abstract screening, which were conducted via the Rayyan web application. Based on predefined eligibility criteria, 4760 records were excluded. The full texts of 72 articles were then assessed for eligibility, resulting in 35 studies failing to meet the inclusion criteria. Ultimately, the current review was comprised of 37 studies.

### Data Extraction

A detailed data extraction sheet was developed in Microsoft Excel by one reviewer (AFD) and validated by two senior reviewers (DBCG and MN). This pre-validated data extraction sheet was used to extract data from the included studies for quality assessment and evidence synthesis. Extracted items included study setting, participant demographics and baseline characteristics, intervention and control group details, study methodology, outcomes, and measurement time points. Two reviewers (AFD and JVD) independently extracted the data, with discrepancies resolved through discussion with a third reviewer (MN). Study authors were contacted via email to obtain any missing information.

The primary outcomes were balance (e.g., Berg Balance Scale, Activities-specific Balance Confidence Scale). and functional mobility (e.g., Timed-Up-and-Go test, Five-Times Sit-to-Stand Test). The secondary outcome was quality of life, assessed using instruments such as the 39-item or 8-item Parkinson’s Disease Questionnaire (PDQ-39; PD Q-8).

### Risk of Bias Assessment of Individual Studies

The risk of bias was independently assessed by two reviewers (AFD and AJ). For randomized controlled trials (RCTs), the second version of the Cochrane Risk of Bias tool (RoB 2) was employed (Sterne et al., 2019), and traffic light plots were generated using the RobVis web application (McGuinness & Higgins, 2021). The quality of quasi-experimental studies, case series, and case reports was evaluated using the respective Joanna Briggs Institute (JBI) critical appraisal tools (Barker et al., 2024; Munn et al., 2020). For single-group pre-post studies, the National Institutes of Health (NIH) study quality assessment tool was utilized.

### Data Analysis

Meta-analyses were conducted using Review Manager (RevMan version 5.2, Cochrane, London, UK). Comparable studies were pooled using a fixed or random-effects model based on heterogeneity. Mean differences or standardized mean differences were calculated for continuous outcomes. Heterogeneity was assessed using the chi-squared test and the I^2^ statistic, with an I^2^ value greater than 50% indicating substantial heterogeneity. Funnel plots were used to identify publication bias. In addition to the quantitative synthesis, a narrative summary of findings from all included studies has been provided.

## Results

### Study Designs and Settings

Randomized controlled trials (n = 14) and one-group pre-post designs (n = 14) were the majority, followed by quasi-experimental studies (n = 5), case series (n = 3), and a case report (n=1). Most studies (n = 29; 78%) originated from high-income countries, particularly the United States of America (n = 9), Italy (n = 5), and Canada (n = 3), with additional contributions from the Netherlands (n = 2), Germany (n = 2), Israel (n = 2) and one study each from Australia, Belgium, Hong Kong, Japan, South Korea, and Sweden. Upper-middle-income countries included Brazil (n = 3), China (n = 1), and Turkey (n = 1). Lower-middle-income countries were represented by India (n = 2), Jordan (n = 1), and South Africa (n = 1). The included studies were published over the span of 2013 to 2025.

### Participant Characteristics

A total of 1,595 participants were included in the studies reviewed. Of these, 607 individuals received home-based TR interventions, while 646 participants were assigned to control or comparator groups. The remaining 342 participants were enrolled in single-group or case-based studies that did not include a control arm. Sample sizes varied considerably, ranging from 19 to 273 participants in parallel-group trials and 15 to 56 participants in single-group designs.

The mean age of participants was 66.3 years, and the average disease duration was 6.6 years. The overall gender distribution showed a predominance of male participants (59.7%). Except for one study that included participants at Hoehn and Yahr (H&Y) stage 4, all other studies recruited individuals within stages 1 to 3, with stage 3 being the most frequently represented. Only 18 studies reported the MDS-UPDRS Part III (motor) scores, with a mean score of 32.18 points. In contrast, the Levodopa Equivalent Daily Dose (LEDD) was reported in 16 studies, with an average dosage of 626 mg/day.

### Intervention Characteristics

Sixteen studies employed multimodal exercise interventions. Collectively, the interventions integrated a range of exercise components, including balance training (n = 25), functional mobility (n = 26), strength training (n = 18), flexibility exercises (n = 17), endurance activities (n = 11), and relaxation techniques (n = 11). Additionally, several studies incorporated recreational or alternative modalities such as exergaming (n = 7), dance (n = 1), and yoga combined with mindfulness meditation (n = 1). Specialized therapeutic approaches included the Lee Silverman Voice Treatment (LSVT) BIG (n = 2) and Baduanjin Qigong (n = 1). Most studies (n = 29; 78%) included a familiarization phase to introduce participants to the TR setup and intervention procedures, as well as to ensure safety throughout the program. In nine studies, caregivers were actively involved to provide supervision and ensure participant safety. Additionally, two studies recommended the presence of caregivers during the intervention sessions.

Smartphones (n = 13), tablets (n = 8), computers (excluding laptops) (n = 6), laptops (n = 3), DVDs (n = 3), Nintendo Wii consoles (n = 2), and Microsoft Kinect systems (n = 2) were among the most frequently utilized devices in the included studies. Virtual reality (VR) technologies were incorporated into home-based TR interventions in six studies, while augmented reality (AR) was employed in one study. Proprietary platforms were used in seven studies to deliver TR interventions. Of these, two studies implemented the Teraplus system, while one study each utilized CuPiD, VRRS, HomeHEAD, VidyoConnect Epic Platform, Salut Digitale, Reality DTx, Smarter Balance System, and START. Software platforms included proprietary or custom-developed mobile health applications, utilized in 10 studies. In addition, several studies employed commercially available platforms such as Zoom (n = 4), Skype (n = 2), WhatsApp (n = 1), and Google Meet (n = 1).

Asynchronous TR delivery modes were employed in 21 studies, while 16 studies utilized synchronous modalities. Videoconferencing served as the primary mode of guidance in 16 studies, and mobile applications were used in 11 studies to facilitate exercise delivery. VR-enhanced exergaming was implemented in six studies, whereas AR-based exergaming was reported in one study. Additionally, three studies employed DVD-guided exercise programs, one study utilized a website-based platform, and another relied on messaging applications or email for guidance. Telephone-based monitoring was the sole method of delivery in nine studies.

The predominant mode of TR content delivery was video based, reported in 29 studies. Thirteen studies employed pre-recorded video content for exercise delivery, while eleven studies utilized live video-based guidance. Additionally, three studies incorporated a combination of both pre-recorded and live video formats.

The control or comparator conditions varied across studies. Fourteen studies employed an active control group. Seven studies compared home-based TR with center-based rehabilitation programs, while six studies used unsupervised home exercise as the comparator. Four studies utilized standard care without any structured exercise intervention. Two studies implemented mixed comparator conditions: one combined home visits with unsupervised home exercise, and another provided only gait advice and training recommendations.

The frequency of intervention sessions varied from once weekly to daily, with an average frequency of approximately three sessions per week. Eighteen studies reported that the exercise programs were individually tailored to participants’ needs, while ten studies described the exercise intensity as moderate. The most employed method for determining training intensity was Borg’s Rating of Perceived Exertion (n = 5). Session durations ranged from 15 to 90 minutes, with an average duration of approximately 43 minutes. The duration of intervention periods spanned from 2 weeks to 12 months, with 8 weeks being most common. Few studies conducted follow-up assessments, which ranged between 1- and 7-months post-intervention.

The findings of all studies are summarized in Table 1.

### Risk of Bias

**Figure 2:**
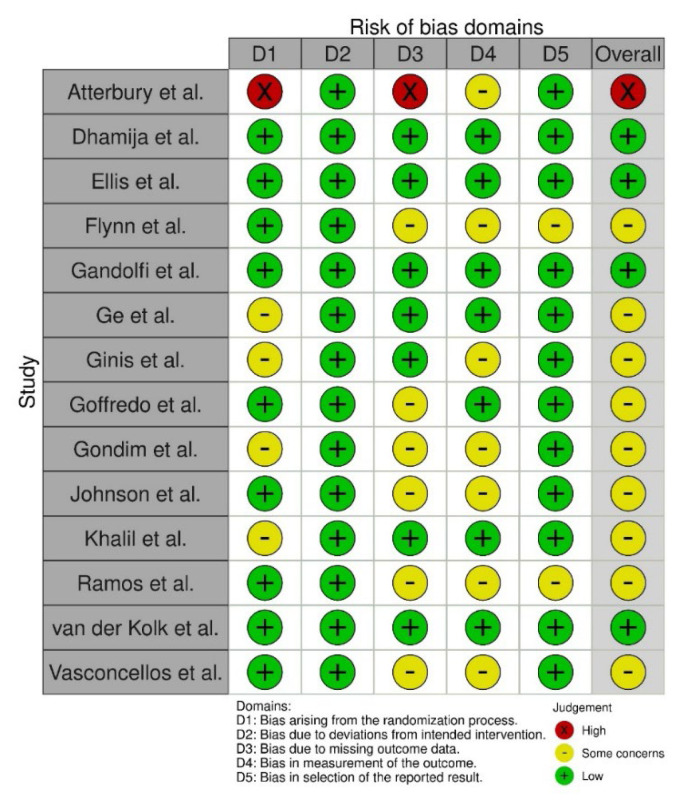
Risk of Bias Assessment

The risk of bias evaluation for all the included RCTs is shown in Figure 2. The study quality assessment for all other included study designs is shown in Appendix B.

## Narrative Synthesis

### Balance

Five RCTs (Gandolfi et al., 2017; Ginis et al., 2016; Goffredo et al., 2023; Johnson et al., 2024; Khalil et al., 2017), two pre-post studies (Kwok et al., 2022; Putzolu et al., 2023), and two case series (Carvalho et al., 2021; Cornejo Thumm et al., 2021) demonstrated improvements in balance. Among these, five studies employed synchronous TR, reporting improvements in the Berg Balance scale (BBS) (Gandolfi et al., 2017; Kwok et al., 2022), Activities-specific Balance Confidence (ABC) scale (Cornejo Thumm et al., 2021; Johnson et al., 2024), and Mini Balance Evaluation Systems Test (MiniBESTest) scores (Carvalho et al., 2021). Four studies utilized asynchronous TR, showing improvements in the MiniBESTest (Ginis et al., 2016; Goffredo et al., 2023; Putzolu et al., 2023), Four Square Step Test (FSST) (Putzolu et al., 2023), and Falls Efficacy Scale-International (FES-I) scores (Khalil et al., 2017). The study by Potzolu et al. (2023) reported improvements in the MiniBEST and FSST at follow-up. In comparisons between TR and center-based rehabilitation, one study (Gandolfi et al., 2017) reported significant gains in BBS scores, whereas another (Johnson et al., 2024) reported notable improvements in ABC scores within a hybrid intervention group combining TR and in-person rehabilitation. Additionally, four studies incorporated mobile applications, which were associated with improvements in MiniBESTest (Ginis et al., 2016; Goffredo et al., 2023; Putzolu et al., 2023), ABC (Johnson et al., 2024), and FSST (Putzolu et al., 2023). Three studies explored TR exergaming: one reported improvement in MiniBESTest scores (Goffredo et al., 2023), whereas two reported enhancements in BBS scores, post-intervention in Gandolfi et al. (2017) and at follow-up in Isernia et al. (2020).

### Functional Mobility

Improvements in functional mobility were reported in two quasi experimental studies (Isernia et al., 2020; Lai et al., 2018), six pre-post studies (Afshari et al., 2024; Cooley Hidecker et al., 2022; Hardeman et al., 2024; Landers & Ellis, 2020; Lavoie et al., 2021; Putzolu et al., 2023), two case series (Carvalho et al., 2021; Cornejo Thumm et al., 2021), and one case report (Chatto et al., 2018). Synchronous TR was used in six studies, resulting in improvements in the Five-Times Sit-To-Stand Test (FTSTS) (Afshari et al., 2024), 30-sec Sit-To-Stand (STS) (Cooley Hidecker et al., 2022), Six-Minute Walk Test (6MWT) (Lai et al., 2018), Ten-Meter Walk Test (10MWT) (Lai et al., 2018), Timed-Up-and-Go test with cognitive task (TUG-C) (Lavoie et al., 2021), and gait speed (Carvalho et al., 2021; Cornejo Thumm et al., 2021). The improvement seen in the TUG-C scores in the study by Lavoie et al. was retained at follow-up. Five studies employed asynchronous TR, reporting gains in Two-Minute Walk Test (2MWT) (Isernia et al., 2020), Timed-Up-and-Go (TUG) test (Chatto et al., 2018; Hardeman et al., 2024), FTSTS (Hardeman et al., 2024), 10MWT (Chatto et al., 2018; Hardeman et al., 2024), gait speed (Hardeman et al., 2024), 30-sec STS (Landers & Ellis, 2020), Short Physical Performance Battery (SPPB) (Putzolu et al., 2023), and the 6MWT (Chatto et al., 2018). No studies comparing TR with center-based rehabilitation reported significant improvements in functional mobility. Mobile applications were used in four studies, which showed improvements in FTSTS (Afshari et al., 2024), 30-sec STS (Landers & Ellis, 2020), 6MWT (Lai et al., 2018), 10MWT (Lai et al., 2018), and SPPB (Putzolu et al., 2023). The improvement seen in the SPPB scores in the study by Potzulo et al. (2023) was retained at follow-up. Three studies investigated TR exergaming; one reported sustained improvement in the 2MWT, which was retained until follow-up (Isernia et al., 2020), whereas the other reported enhancements in the TUG (Chatto et al., 2018; Hardeman et al., 2024), FTSTS (Hardeman et al., 2024), 6MWT (Chatto et al., 2018), 10MWT (Chatto et al., 2018; Hardeman et al., 2024), and gait speed (Hardeman et al., 2024).

### Quality of Life

Two RCTs (Ginis et al., 2016; Gondim et al., 2017), six pre-post studies (Ahn et al., 2024; Fırat et al., 2023; Landers & Ellis, 2020; Lavoie et al., 2021; Okusa et al., 2025; Walton et al., 2022), and a case series (Carvalho et al., 2021) reported improvements in quality of life. Five studies utilized synchronous TR, resulting in improvements in the summary index scores of the Parkinson’s Disease Questionnaire (PDQ-39) (Carvalho et al., 2021; Fırat et al., 2023; Lavoie et al., 2021; Okusa et al., 2025; Walton et al., 2022), whereas three studies reported improved mobility subscale scores (Carvalho et al., 2021; Fırat et al., 2023; Walton et al., 2022). The improved PDQ-39 summary index scores were retained at follow-up in the study by Lavoie et al. (2021). Three studies using asynchronous TR reported enhancements in the Short Form Health Survey (SF-36) physical health score (Ginis et al., 2016), PDQ-39 summary index (Ahn et al., 2024; Gondim et al., 2017), and the short form Parkinson’s Disease Questionnaire (PDQ-8) (Landers & Ellis, 2020). Among studies comparing TR with center-based rehabilitation, one demonstrated significant improvements in PDQ-39 summary scores (Gondim et al., 2017). Three studies employing mobile applications reported improvements in the SF-36 (Ginis et al., 2016), PDQ-39 (Ahn et al., 2024), and PDQ-8 scores (Landers & Ellis, 2020). Notably, none of the studies utilizing TR exergaming reported improvements in quality of life.

### Safety of Home-based TR for PD

Only eight studies reported adverse events related to TR interventions (Flynn et al., 2020; Gandolfi et al., 2017; Ge et al., 2024; Ginis et al., 2016; Hardeman et al., 2024; Kwok et al., 2022; Pastana Ramos et al., 2023; Putzolu et al., 2023; van der Kolk et al., 2019; Vasconcellos et al., 2023). The most frequently reported events were musculoskeletal pain (Flynn et al., 2020; Ge et al., 2024; Pastana Ramos et al., 2023; van der Kolk et al., 2019; Vasconcellos et al., 2023) and fatigue (Ge et al., 2024; Hardeman et al., 2024; Pastana Ramos et al., 2023; Putzolu et al., 2023; van der Kolk et al., 2019), each of which was documented in five studies. Less commonly reported events, each noted in a single study, included dyspnea (van der Kolk et al., 2019), muscle soreness (Flynn et al., 2020), joint soreness (Flynn et al., 2020; van der Kolk et al., 2019), dizziness (van der Kolk et al., 2019), and near falls (Hardeman et al., 2024; Kwok et al., 2022). Serious adverse events, such as falls, syncope, palpitations, (van der Kolk et al., 2019) and Achilles tendon injury (Flynn et al., 2020), were also reported by one study.

## Meta-analysis

### Balance

**Figure 3:**
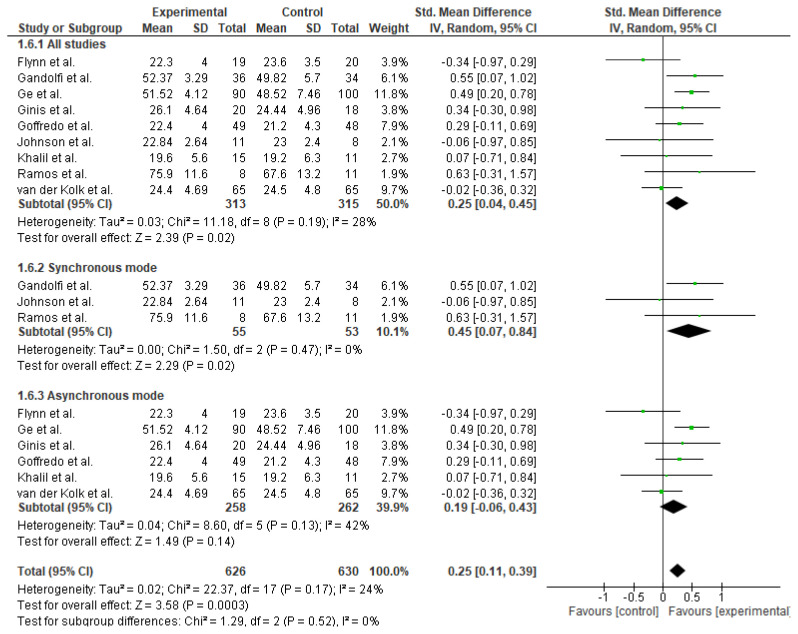
Effect of Home-based TR on Balance Ability Overall

Meta-analysis of nine studies (n = 628) indicated that TR led to a small but statistically significant improvement in overall balance (Standardized Mean Difference [SMD] = 0.25; 95% Confidence Interval [CI]: 0.04 to 0.45; p = 0.02), with low heterogeneity (I^2^ = 28%). A subgroup analysis based on the mode of TR revealed that the synchronous format led to a moderate and statistically significant improvement in overall balance performance (SMD = 0.45; 95% CI: 0.07 to 0.84; p = 0.02). See Figure 3

**Figure 4:**
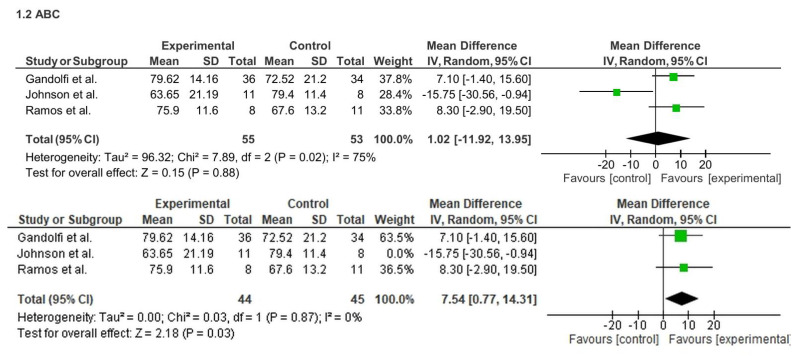
Effect of Home-based TR on Individual Balance ability Measures

When examining balance ability, six studies (n = 525) using the MiniBESTest found no significant difference between TR and control groups (Mean Difference [MD] = 0.17; 95% CI: −0.35 to 0.69; p = 0.53; I^2^ = 0%), whereas two studies (n = 260) using the BBS reported a significant improvement (MD = 2.83; 95% CI: 1.49 to 4.17; p < 0.0001; I^2^ = 0%). See Figure 4.

**Figure 5:**
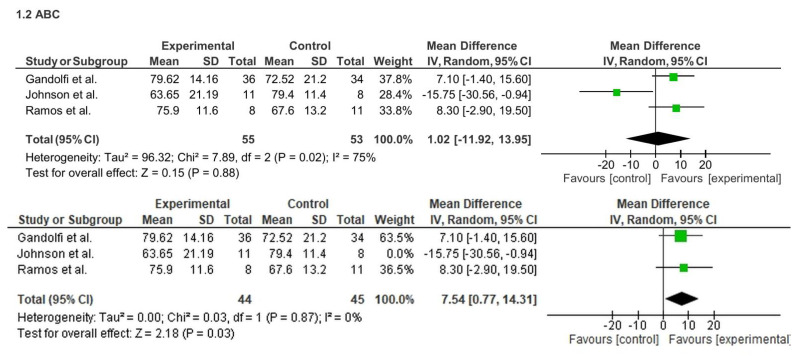
Effect of Home-based TR on ABC Scale

Regarding balance confidence, three studies (n = 108) initially showed no significant effect on the ABC scale (MD = 1.02; 95% CI: −11.92 to 13.95; p = 0.88), with high heterogeneity (I^2^ = 75%). However, sensitivity analysis excluding one outlier study (Johnson et al., 2024) revealed a significant improvement (MD = 7.54; 95% CI: 0.77 to 14.31; p = 0.03; I^2^ = 0%). See Figure 5.

**Figure 6:**
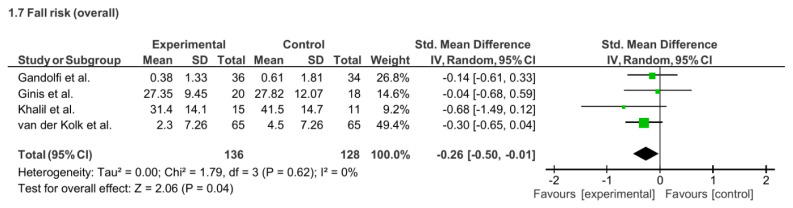
Effect of Home-based TR on Fall Risk (Overall)

Regarding balance confidence, three studies (n = 108) initially showed no significant effect on the ABC scale (MD = 1.02; 95% CI: −11.92 to 13.95; p = 0.88), with high heterogeneity (I^2^ = 75%). However, sensitivity analysis excluding one outlier study (Johnson et al., 2024) revealed a significant improvement (MD = 7.54; 95% CI: 0.77 to 14.31; p = 0.03; I^2^ = 0%). See Figure 6

**Figure 7:**
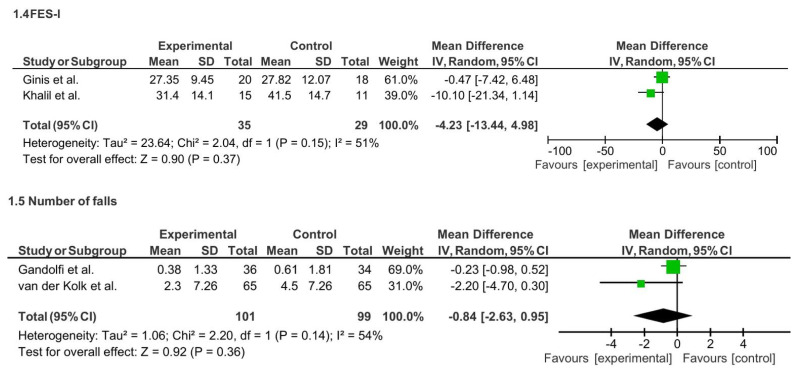
Effect of Home-based TR on Individual Fall Risk Measures

In terms of fall risk, four studies (n = 264) demonstrated a small but significant reduction (SMD = −0.26; 95% CI: −0.50 to − 0.01; p = 0.04; I^2^ = 0%). However, two studies (n = 64) using the FES-I. found no significant difference (MD = −4.23; 95% CI: − 13.44 to 4.98; p = 0.37; I^2^ = 51%), and two studies (n = 200) assessing the number of falls also reported no significant change (MD = −0.84; 95% CI: −2.63 to 0.95; p = 0.36; I^2^ = 54%). See Figure 7.

### Functional Mobility

**Figure 8:**
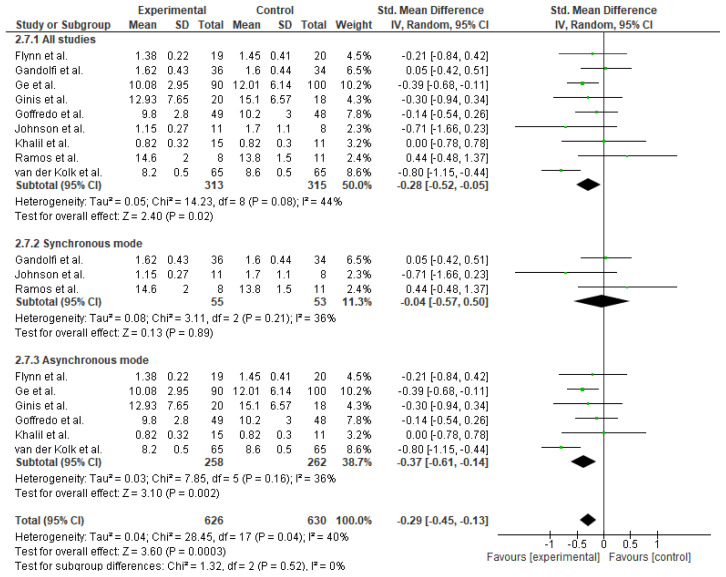
Effect of Home-based TR on Functional Mobility (Overall)

Meta-analysis of nine studies (n = 628) assessing functional mobility revealed a small but statistically significant improvement favoring TR intervention (SMD = −0.28; 95% CI: −0.52 to −0.05; p = 0.02), with moderate heterogeneity (I^2^ = 44%). A subgroup analysis for mode of TR revealed a statistically significant small-to-moderate effect favoring the asynchronous TR intervention (SMD = −0.37; 95% CI: −0.61 to −0.14; p = 0.002), with low-to-moderate heterogeneity (I^2^ = 36%). See Figure 8.

**Figure 9:**
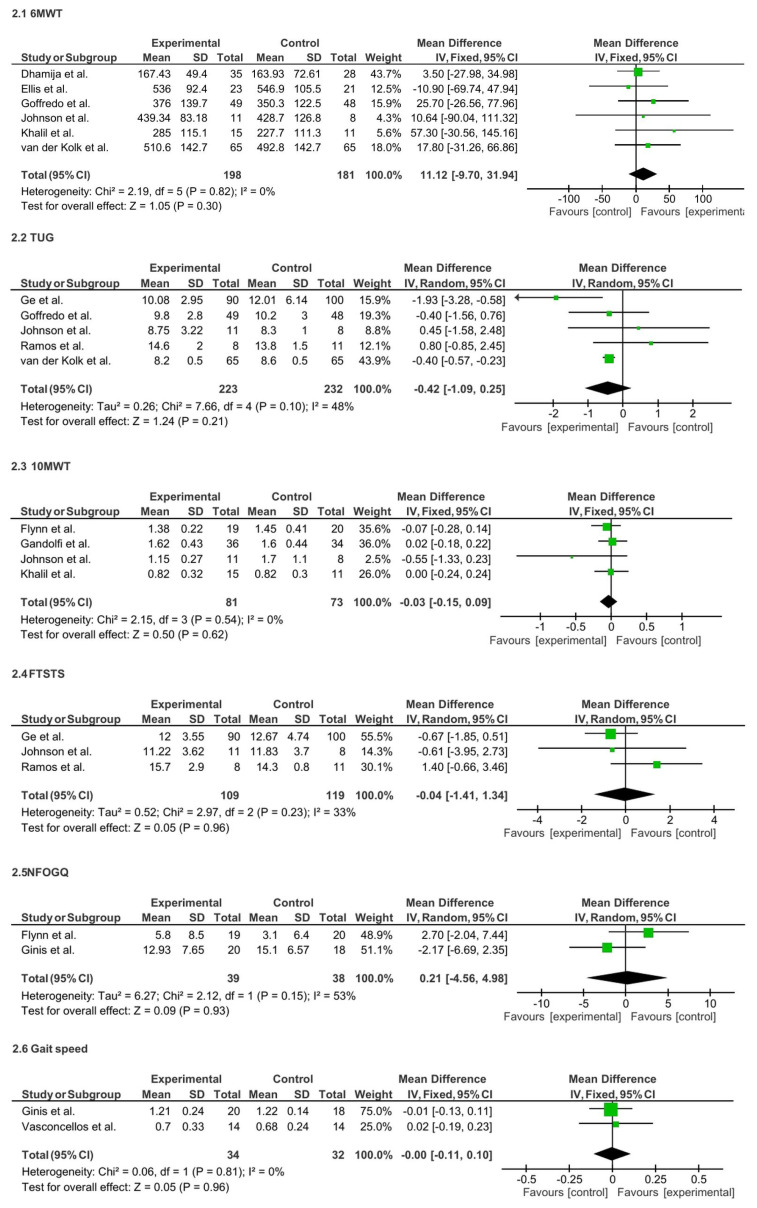
Effect of Home-based TR on Individual Functional Mobility Measures

Analysis of specific functional mobility measures showed that six studies (n = 379) using the 6MWT demonstrated a non-significant trend favoring TR (MD = 11.12; 95% CI: −9.70 to 31.94; p = 0.30; I^2^ = 0%). Similarly, five studies (n = 455) using the TUG test reported a non-significant trend in favor of TR (MD = −0.42; 95% CI: −1.09 to 0.25; p = 0.21), with moderate heterogeneity (I^2^ = 48%). Four studies (n = 154) assessing the 10MWT found no significant difference (MD = −0.03; 95% CI: − 0.15 to 0.09; p = 0.62; I^2^ = 0%). Three studies (n = 228) evaluating the FTSTS also showed no significant difference (MD = − 0.04; 95% CI: −1.41 to 1.34; p = 0.96; I^2^ = 33%). Two studies (n = 77) using the NFOGQ reported no significant change (MD = 0.21; 95% CI: −4.56 to 4.98; p = 0.93; I^2^ = 53%). Lastly, two studies (n=66). assessing gait speed found no significant difference (MD = −0.00; 95% CI: −0.11 to 0.10; p = 0.96; I^2^ = 0%). See Figure 9.

### Quality of Life

**Figure 10:**
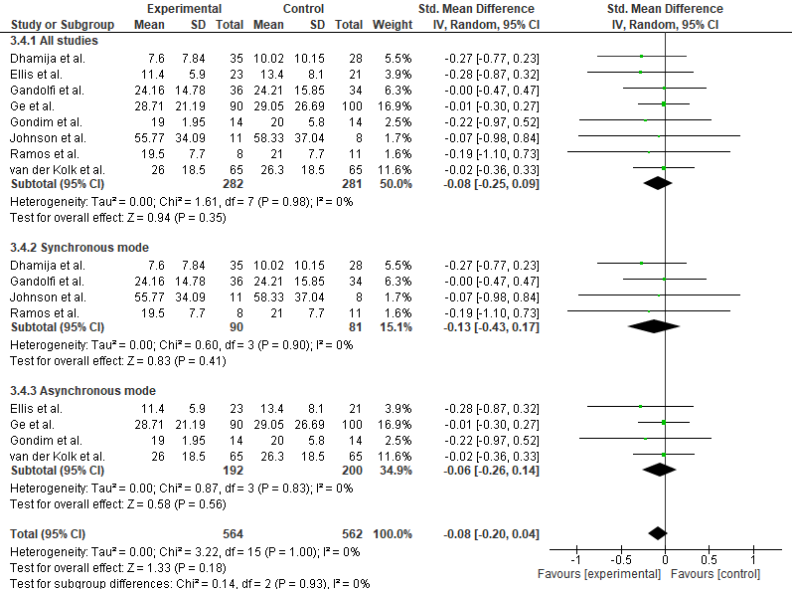
Effect of Home-based TR on Quality of Life (Overall)

Meta-analysis of eight studies (n = 563) evaluating quality of life outcomes revealed a non-significant trend favoring TR intervention (SMD = −0.08; 95% CI: −0.25 to 0.09; p = 0.35)., with no heterogeneity (I^2^ = 0%). Subgroup analysis revealed no favorability to either mode of TR. See Figure 10.

**Figure 11:**
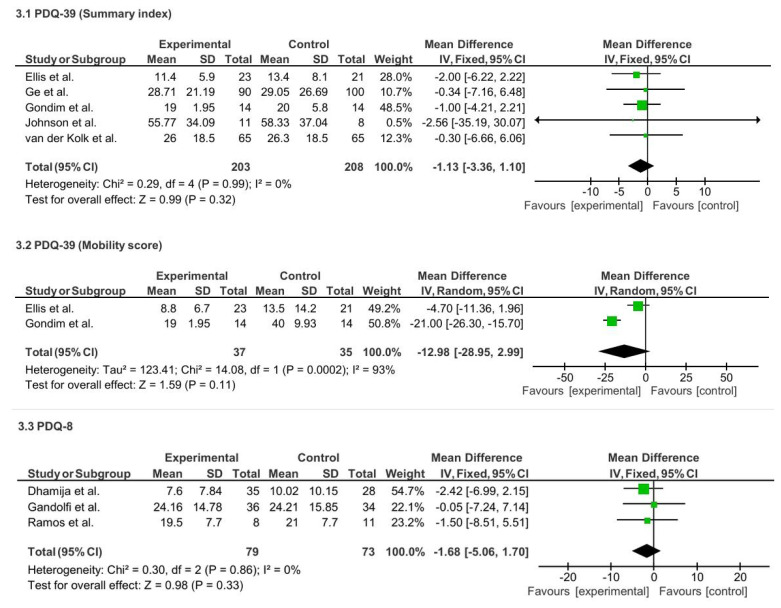
Effect of Home-based TR on Individual Quality of Life Measures

Five studies (n = 411) assessing the PDQ-39 Summary Index also showed a non-significant trend in favor of TR (MD = − 1.13; 95% CI: −3.36 to 1.10; p = 0.32; I^2^ = 0%). Two studies (n = 72) evaluating the PDQ-39 Mobility subscale reported a non-significant trend favoring TR (MD = −12.98; 95% CI: −28.95 to 2.99; p = 0.11), though with high heterogeneity (I^2^ = 93%). Similarly, three studies (n = 152) using the PDQ-8 found no significant difference (MD = −1.68; 95% CI: −5.06 to 1.70; p = 0.33; I^2^ = 0%). See Figure 11.

## Discussion

This systematic review and meta-analysis were conducted to assess the current evidence on the effectiveness of home-based TR in enhancing balance, functional mobility, and quality of life in PwPD.

### Balance

The meta-analysis demonstrated a small but statistically significant improvement in balance among PwPD. These findings are consistent with recent systematic reviews that evaluated the effect of TR in PD (Park & Lee, 2024; Vellata et al., 2021). Both synchronous and asynchronous TR modes improved balance and confidence, but synchronous TR showed significantly greater benefits in subgroup analyses, consistent with findings from a recent scoping review (Silva-Batista et al., 2024). Furthermore, it has been established that supervised balance training programs have been shown to have superior results to unsupervised, self-guided exercise among older adults (Lacroix et al., 2015). Our findings affirm this in the TR context, where videoconference-supervised VR-based exergaming yielded superior balance outcomes compared to center-based rehabilitation, in line with prior systematic reviews (Chuang et al., 2022; Kwon et al., 2023). A hybrid model combining in-person and virtual sessions resulted in greater improvements in balance confidence than center-based programs alone (Gopal et al., 2022; Langer et al., 2021). While few studies reported follow-up data, early evidence suggests potential long-term benefits, as also indicated in studies across other populations (Uche-Okoye et al., 2023; Zhong et al., 2023).

The meta-analysis also indicated a slight reduction in fall risk, despite individual studies not consistently reporting significant improvements. This was a rarely reported outcome in the studies included. Given that fall risk is a critical concern in this population, especially in the context of remote interventions without direct supervision, it should be a key consideration in the design of future TR programs.

### Functional Mobility

The meta-analysis indicated a small but statistically significant improvement in functional mobility, even though individual outcome measures did not consistently reach statistical significance. Given the variability in findings, the clinical significance of these improvements remains uncertain and should be interpreted with caution. Despite this variability, most metrics showed positive trends favoring TR, in agreement with prior reviews (Vellata et al., 2021). Unlike balance outcomes, asynchronous TR was more frequently associated with improvements in mobility, possibly due to the limited number of synchronous TR studies in this domain. VR-enhanced exergaming also demonstrated potential, with improvements observed across several mobility metrics, some of which were sustained at follow-up. Multiple reviews have affirmed that VR exergaming is effective in improving mobility in PD. Importantly, no study comparing home-based TR with center-based rehabilitation reported superior outcomes for the former. This was also reported in a review comparing the effects of home and center-based exercise for PD (Flynn et al., 2019). Nevertheless, inherent advantages of in-person rehabilitation, such as access to specialized equipment, direct therapist supervision, and larger training spaces, support more intensive and tailored interventions and might yield better outcomes. A hybrid model combining TR and center-based rehabilitation needs to be investigated to improve gait.

### Quality of Life

The meta-analysis revealed a non-significant trend favoring TR, making the evidence for its impact on quality of life less conclusive. While some individual studies reported improvements, these findings were neither consistent nor statistically significant. Both synchronous and asynchronous modes of TR were associated with improvement in quality of life. VR-enhanced exergaming did not exhibit any notable improvement in this domain, as noted in another systematic review (Kwon et al., 2023). It has been reported in literature that exercise-based interventions of longer intervention periods are required to cause a meaningful improvement in quality of life in PwPD (Chen et al., 2020; Yang et al., 2023). Hence, it could be inferred that the lack of effect of home-base TR on quality of life may be a reflection of the relatively shorter intervention period in the included studies which may have provided insufficient time for the emergence of improvements in quality of life. Such findings underline the need for future research which is focused on the optimization of home-based TR programs for effectively improving quality of life in PwPD. Studies implementing sufficiently long intervention periods with follow-up could potentially provide adequate time required for improvements in quality of life to be captured.

### Safety of TR

Home-based TR is generally safe for PwPD. Most of the reported adverse events were mild, which included musculoskeletal pain, while some studies reported post-exercise fatigue as well. In contrast, serious adverse events were rare and notably were reported by studies wherein direct remote supervision was not carried out. This highlights the significance of integrating robust mechanisms for monitoring TR participants to ensure their safety, as well as designing customizable TR protocols which place emphasis on participant safety while being easy to follow. The risk of fall and the overall safety of home-based TR could be mitigated by ensuring real-time supervision wherever feasible and incorporating automated safety mechanisms as well as a participant feedback system (Gutierrez-Arias et al., 2025).

### Strengths of the review

The strength of this review is that it encompasses a wide representation of interventional study designs which include randomized controlled trials, quasi-experimental studies, pre-post studies, and case series. This provides a broader representation of the current level of evidence on home-based TR for balance, functional mobility, and quality of life in PwPD.

#### Limitations

There was significant heterogeneity in the contents of the TR intervention program, study durations, and TR delivery mode among other characteristics which might limit the generalizability of the findings to TR at large due to unequal representation. Due to limited number of studies reporting follow-up assessment of outcomes, the long-term effects of home-based TR remain inconclusive for PwPD. In addition, the inconsistent reporting of several key factors like digital literacy of participants, accessibility to technology, and involvement of the caregiver in the intervention delivery will also limit the generalizability of these findings among PwPD from lower socioeconomic backgrounds.

#### Future implications

There is considerable variability in the delivery methods of TR, which may contribute to inconsistent outcomes across studies. Future research should adopt more rigorous and standardized study protocols to yield more conclusive evidence. Moreover, the long-term effectiveness of home-based TR remains underexplored and requires further investigation with adequate intervention period and longer follow-up duration. Due to a relative dearth of literature that investigates the effect of a hybrid TR model, this could be a promising area for future exploration.

Moreover, the application of TR in low-resource settings is an area of significant untapped potential. Individuals from lower socioeconomic backgrounds often face considerable barriers to accessing traditional, center-based rehabilitation services. As such, they may benefit most from affordable and accessible TR interventions. Future research should focus on designing and evaluating low-cost, adaptable TR solutions that are tailored to the specific needs and constraints of underserved populations, thereby promoting equity in access to rehabilitation care. Similarly, those with advanced PD and greater mobility restrictions are excluded from TR research, which could be another area of interest.

## Conclusion

Home-based telerehabilitation appears to be an effective and safe approach for improving balance and functional mobility in PwPD. However, its impact on quality of life remains limited, potentially due to the relatively short duration of interventions in most studies. The overall feasibility of telerehabilitation across diverse settings supports its broader implementation, particularly when integrated with structured supervision and personalized delivery models. Future research should focus on optimizing program duration, enhancing long-term outcomes, and addressing the needs of underserved and advanced-stage PwPD.

## Figures and Tables

**Table 1:** Findings from Studies

S no	Study, Year, Country	Study design	Sample size	Participant characteristics [Age & Disease duration in years as Mean (SD); H&Y stage as Range]	Type of TR	Intervention	Dosage of TR intervention	Intervention period	Follow-up period	Control/Comparator	Outcome measures	Findings
1	Atterbury & Wellman, 2017 South Africa	Randomized controlled trial	Total: 40 IG: 16 CG: 24	Age: 65 (7.6) Disease duration: 5 (7.1) H&Y stage: 1.5 to 3	Asynchronous	DVD-guided exercise. Caregiver supervised all sessions.	Frequency: 3 x / week, Intensity: Individually tailored Time: 40 – 60 min Type: Balance; Flexibility; Relaxation	8 weeks	-	In-clinic balance training	Balance: ABC Functional mobility: ITUG	ABC scores were significantly improved in the therapist-supervised group.
2	Dhamija et al., 2025 India	Randomized controlled trial	Total: 64 IG: 36 CG: 28	Age: 61.7 (10.4) Disease duration: 4.6 (3.9) H&Y stage: 1 to 2.5	Synchronous	WhatsApp videoconference-guided exercise delivered via smartphone (tapered frequency) and unsupervised home exercise (5 x / week)	Frequency: 1 x / week (first 4 weeks); 1 x / 2 weeks (next 8 weeks) Intensity: Moderate to Somewhat severe on modified Borg Dyspnea scale 3 – 4) Time: 30 min Type: Balance; Strength; Endurance; Flexibility; Relaxation	12 weeks	-	In-clinic exercise training (tapered frequency like TR group) and unsupervised home exercise (5 x / week). Exercises were the same as TR group.	Balance: FRT Functional mobility: 6MWT Quality of life: PDQ-8	There was no difference in any outcomes between the groups
3	Ellis et al., 2019 United States	Randomized controlled trial	Total: 51 IG: 26 CG: 25	Age: 64.1 (9.5) Disease duration: 4.8 (3.1) H&Y stage: 1 to 3	Asynchronous	Wellpepper mobile application-guided exercise delivered via tablet and pedometer-tracked walking	Frequency: 3 x / week, Intensity: Individually tailored Time: Not mentioned Type: Strength; Flexibility; Functional mobility	12 months	-	Standard home exercise program and pedometer-tracked walking	Functional mobility: 6MWT Quality of life: PDQ-39 (Summary index and Mobility score)	There was no difference in any outcomes between the groups
4	Flynn et al., 2020 Australia	Randomized controlled trial	Total: 40 IG: 20 CG: 20	Age: 72 (6.9) Disease duration: 5 (4.9) H&Y stage: 1 to 3	Asynchronous	PTEx website-guided unsupervised home exercise after 5 weeks of in-clinic exercise. Telephone monitoring was done on 7th and 9th week.	Frequency: 3 x / week Intensity: Individually tailored Time: 45 – 60 min Type: Balance; Functional mobility; Relaxation	10 weeks	-	In-clinic group-based exercises similar to the TR group.	Balance: MiniBESTest Functional mobility: 10MWT; NFOGQ	There was no difference in any outcomes between the groups
5	Gandolfi et al., 2017 Italy	Randomized controlled trial	Total: 76 IG: 38 CG: 38	Age: 68.6 (8.4) Disease duration: 6.8 (3.8) H&Y stage: 2.5 & 3	Synchronous	Skype videoconference-guided TeleWii training (Nintendo Wii exergaming) via laptop computer. Caregiver supervised all sessions.	Frequency: 3 x / week Intensity: Individually tailored Time: 50 min Type: Balance; Flexibility	7 weeks	1 month	In-clinic sensory integration balance training	Balance: BBS; ABC; DGI; Number of falls Functional mobility: 10MWT Quality of life: PDQ-8	BBS scores were significantly improved in the TR group
6	Ge et al., 2024 China	Randomized controlled trial	Total: 190 IG: 90 CG: 100	Age: 70 (5.9) Disease duration: Not mentioned H&Y stage: 1 to 3	Asynchronous	An unspecified mobile application-guided exercise program delivered via smartphone. Caregiver supervision was recommended.	Frequency: 5 x / week Intensity: Low to Moderate on Borg RPE scale (11 – 14) Time: 40 – 60 min Type: Balance; Strength; Functional mobility; Flexibility; Endurance	4 weeks	-	Supervised home exercise (3 x / week) and unsupervised home exercise (2 x / week), same as the TR group	Balance: BBS Functional mobility: TUG; FTSTS Quality of life: PDQ-39	Overall, there was no difference in any outcomes between the groups. However, in older participants (70 years and over) there was significant improvement s in BBS and TUG between groups.
7	Ginis et al., 2016 Belgium; Israel	Randomized controlled trial	Total: 40 IG: 22 CG: 18	Age: 66.7 (8) Disease duration: 11.1 (6.4) H&Y stage: 2 & 3	Asynchronous	CuPiD system-mediated exercise training. It involves a mobile application and sensors which provide auditory biofeedback for gait and cues for FOG-training through a smartphone.	Frequency: At least 3 x / week Intensity: Moderate (ACSM guidelines) Time: 30 min (Additional 30 min for those with FOG) Type: Functional mobility	6 weeks	1 month	Gait advice and training recommendations were provided	Balance: MiniBESTest; FSST; FES-I Functional mobility: Gait speed (single / dual task); 2MWT; NFOGQ Quality of life: SF-36 (Total; Physical health)	MiniBESTest and SF-36 physical health scores were significantly improved in the CuPiD group.
8	Goffredo et al., 2023 Italy	Randomized controlled trial	Total: 104 IG: 54 CG: 51	Age: 68 (6.1) Disease duration: 5 (4.9) H&Y stage: 1 to 2.5	Asynchronous	VRRS-tablet system-guided non-immersive VR exergaming intervention	Frequency: 3 – 5 x / week (30 sessions) Intensity: Moderate Time: 45 min Type: Functional mobility; Strength; Flexibility; Balance	6 – 10 weeks	-	Unsupervised home exercise plan includes balance and functional mobility exercises.	Balance: MiniBESTest Functional mobility: TUG; 6MWT	MiniBESTest scores were significantly improved in the TR group
9	Gondim et al., 2017 Brazil	Randomized controlled trial	Total: 28 IG: 14 CG: 14	Age: 65 (7.5) Disease duration: 4.5 (2.5) H&Y stage: 1 to 3	Asynchronous	Exercises through the Pro-Parkinson’s program booklet. Individualized guidance was given and telephone monitoring was done weekly.	Frequency: 3 x / week Intensity: Not mentioned Time: 60 min Type: Flexibility; Functional mobility; Balance; Strength	12 weeks	-	In-clinic group-based Pro-PD program (2x /month;40 min duration) + unsupervised home exercises as performed by TR group, but without telephone monitoring or guidance.	Quality of life: PDQ-39 (Summary index and Mobility score)	PDQ-39 scores were significantly improved in the TR group
10	Johnson et al., 2024 United States	Randomized controlled trial	Total: 20 IG 1: 6 IG 2: 6 CG: 8	Age: 72.6 (5.3) Disease duration: 3 (4.12) H&Y stage: 1.5 to 2.5	Synchronous	WizeCare mobile/web application videoconference-guided exercise program delivered via any device of participant’s choice. IG 1 received only TR, while IG 2 received TR and in-clinic exercise training. Caregiver supervised all sessions.	Frequency: 1 x / week Intensity: Not mentioned Time: 60 min Type: Not mentioned	4 weeks	-	In-clinic conventional PT	Balance: MiniBESTest; ABC Functional mobility: TUG; TUG-C; FTSTS; 10MWT; 6MWT Quality of life: PDQ-39	ABC scores were significantly improved in the hybrid (TR & in-clinic) group compared to usual care. There was no difference in any other outcomes between TR, hybrid, and in-clinic groups.
11	Khalil et al., 2017 Jordan	Randomized controlled trial	Total: 30 IG: 16 CG: 14	Age: 59.4 (14.4) Disease duration: 7.7 (5.4) H&Y stage: 1 to 3	Asynchronous	DVD-guided exercise and self-monitored walking. Telephone monitoring was done weekly.	Frequency: 3 x / week (DVD); 1 x / week (Walking) Intensity: Moderate (3 – 4 on the Borg CR10 RPE scale) Time: 45 min Type: Strength; Balance; Endurance; Functional mobility; Relaxation; Flexibility	8 weeks	-	Standard care (no exercise intervention)	Balance: MiniBESTest; FES-I Functional mobility: 10MWT; 6MWT; 30-sec STS	FES-I scores were significantly improved in the DVD group
12	Ramos et al., 2023 Brazil	Randomized controlled trial	Total: 19 IG: 8 CG: 11	Age: 59.3 (12.2) Disease duration: 5.3 (5.7) H&Y stage: 1 to 2	Synchronous	WhatsApp or Google Meet videoconference-guided exercise delivered via any device of the participant’s choice. Caregiver supervised all sessions.	Frequency: 3 x / week Intensity: Moderate Time: 60 min Type: Functional mobility; Strength; Balance; Flexibility; Relaxation	4 weeks	2 months	Unsupervised home exercise plan that included the same exercises as the TR group via booklet	Balance: ABC Functional mobility: TUG; FTSTS Quality of life: PDQ-8	There was no difference in any outcomes between the groups
13	van der Kolk et al., 2019 Netherlands	Randomized controlled trial	Total: 130 IG: 65 CG: 65	Age: 59.3 (8.7) Disease duration: 3.76 (1.4) H&Y stage: 1 & 2	Asynchronous	VR-enhanced stationary cycle (exergaming) and motivational mobile application delivered through a tablet	Frequency: At least 3 x / week Intensity: 50 – 70% of HRR Time: 30 – 45 min Type: Endurance	6 months	-	Unsupervised home exercise plan that includes stretching and relaxation exercises.	Balance: Number of falls; MiniBESTest Functional mobility: 6MWT; TUG Quality of life: PDQ-39	There was no difference in any outcomes between the groups
14	Vasconcellos et al., 2021 Brazil	Randomized controlled trial	Total: 28 IG: 14 CG: 14	Age: 65.6 (8.1) Disease duration: 6 (4.2) H&Y stage: 2 & 3	Asynchronous	Trunk-exercise program delivered via smartphone messaging application or email through booklet and videos. Caregiver supervised all sessions. Telephone monitoring was done once a day.	Frequency: Daily (3 x / day) Intensity: Individually tailored Time: Not mentioned Type: Strength; Functional mobility; Relaxation	3 weeks	1 month	Unsupervised home exercise plan that includes relaxation, stretching, and functional mobility exercises delivered via booklet and videos.	Balance: Bertec force platform Functional mobility: Qualisys movement analysis; Gait speed	There was no difference in any outcomes between the groups
15	Horin et al., 2019 United States	Quasi-experimental	Total: 37 IG: 17 CG: 20	Age: 64.1 (8.5). Disease duration: 6.3 (4.9). H&Y stage: 2 & 3	Asynchronous	Beats Medical Parkinsons Treatment mobile application-guided exercise delivered via smartphone	Frequency: Daily Intensity: Not mentioned Time: 30 min Type: Functional mobility	12 weeks	-	Standard care (no exercise intervention)	Functional mobility: Gait speed	There was no difference in any outcomes between the groups
16	Isernia et al., Italy 2020	Quasi-experimental	Total: 31 IG: 11 CG: 20	Age: 66.8 (9.1) Disease duration: Not mentioned H&Y stage: 1 & 2	Asynchronous	HomeHEAD intervention - VR-based exergaming delivered via Microsoft Kinect and Leap Motion after undergoing 4 weeks of ClinicHEAD. Telephone monitoring was done periodically.	Frequency: 5 x / week Intensity: Individually tailored Time: 45 min Type: Balance; Endurance; Functional mobility; Strength	12 weeks	7 months	Standard care (no exercise intervention)	Balance: BBS Functional mobility: 10MWT; 2MWT Quality of life: SF-12; PANAS	The 2MWT scores were significantly improved in the TR group. This was retained at follow-up with significant improvement in BBS scores as well.
17	Lai et al., 2020 United States	Quasi-experimental	Total: 20 IG: 10 CG: 10	Age: 67.1 (9.4) Disease duration: 7 (4.5) H&Y stage: 1 to 3	Synchronous	Custom mobile application videoconference-guided exercise delivered via tablet and wearable HR monitor for feedback	Frequency: 3 x / week Intensity: 40 – 60% of HRR; 1 – 5 lb weight cuff Time: 165 min per week Type: Endurance; Strength	8 weeks	-	Unsupervised exercise using the same application	Functional mobility: 6MWT; 10MWT	The 6MWT scores were much higher in the TR group. There was a smaller improvement in 10MWT scores in the TR group.
18	Lutzow et al., 2023 Germany	Quasi-experimental	Total: 32 IG: 16 CG: 16	Age: 64.8 (7.8) Disease duration: 8 (6) H&Y stage: 2 & 2.5	Asynchronous	PatientConcept mobile application-guided exercise delivered via smartphone.	Frequency: 3 – 5 x / week Intensity: Not mentioned Time: 20 – 30 min Type: Balance; Flexibility; Strength; Functional mobility	26 weeks	-	Standard care (no exercise intervention)	Functional mobility: TUG; 2MWT Quality of life: PDQ-39 (Summary index & Mobility score)	There was no difference in any outcomes between the groups
19	Wagner et al., 2022 Germany	Quasi-experimental	Total: 273 IG: 123 CG: 150	Age: 66.2 (9.4) Disease duration: 8 (5.5) H&Y stage: 2 to 3	Asynchronous	ParkProTrain mobile application-guided exercise delivered via tablet after 3-weeks of in-patient care. Telephone monitoring was done every three weeks.	Frequency: At least 3 x / week Intensity: Individually tailored Time: Not mentioned Type: Endurance; Strength; Balance	9 months	-	In-clinic conventional PT	Balance: FES-I Quality of life: PDQ-8	There was no difference in any outcomes between the groups
20	Afshari et al., 2024 United States	One group pre-post	15	Age: 67 (6.6) Disease duration: 8 (2.2) H&Y stage: 2 & 3	Synchronous	VidyoConnect Epic platform videoconference-guided exercise was delivered via a tablet mounted on a stand with wheels. Caregiver supervised all sessions.	Frequency: 2 x / week Intensity: Not mentioned Time: 30 min Type: Balance; Functional mobility	10 weeks	-	-	Balance: MiniBESTest; ABC; FES-I Functional mobility: FTSTS; TUG; TUG-C; 10MWT; 2MWT Quality of life: PDQ-39	FTSTS scores were significantly improved
21	Ahn et al., 2024 South Korea	One group pre-post	41	Age: 64.1 (8.3) Disease duration: 1.9 (2.9) H&Y stage: 1 to 3	Asynchronous	PDexercise mobile-application or web-application-guided exercise delivered via smartphone	Frequency: At least 5 x / week Intensity: Moderate intensity Time: 15 – 20 min Type: Functional mobility; Flexibility; Strength	2 weeks	-	-	Quality of life: PDQ-39 (Summary index and Mobility score)	PDQ-39 summary index scores were significantly improved
22	Bianchini et al., Italy 2022	One group pre-post	23	Age: 64. 2 (8.9) Disease duration: 6.5 (3.8) H&Y stage: 1 to 2.5	Synchronous	Salut Digitale videoconference-guided exercise and unsupervised home exercise (2 x / week). Caregiver supervised all sessions.	Frequency: 1 x / week Intensity: Individually tailored Time: 40 – 60 min Type: Functional mobility; Balance	5 weeks	1 month	-	Quality of life: PDQ-39	There was no improvement
23	Firat et al., 2023 Turkey	One group pre-post	15	Age: 63.13 (9.89) Disease duration: 3.67 (4.29) H&Y stage: 1 to 3	Synchronous	Videoconference-guided LSVT-BIG (unspecified platform and device)	Frequency: 4 x / week Intensity: At least 80% of maximum effort Time: 1 hour Type: Functional mobility	4 weeks	-	-	Quality of life: PDQ-39 (Summary index and Mobility score)	PDQ-39 summary index and mobility scores were significantly improved
24	Garg et al., 2021 India	One group pre-post	22	Age: 61.7 (6.7) Disease duration: 4.9 (3.7) H&Y stage: Not mentioned	Synchronous	An unspecified mobile application videoconference-guided exercise delivered via smartphone (tapered frequency) and unsupervised home exercise (5 x / week)	Frequency: 1 x / week (first 4 weeks); 1 x / 2 weeks (next 8 weeks) Intensity: Moderate to somewhat severe on modified Borg Dyspnea scale (3 – 4) Time: 30 min Type: Balance; Strength; Endurance; Flexibility; Relaxation	12 weeks	-	-	Quality of life: PDQ-8	There was no improvement
25	Hardeman et al., 2024 Netherlands	One group pre-post	24	Age: 65.5 (6.8) Disease duration: 8.6 (4.1) H&Y stage: 2 & 2.5	Asynchronous	Reality DTx exergaming intervention delivered via Microsoft Leap or HoloLens AR glasses. Telephone monitoring was done weekly.	Frequency: At least 5 x / week Intensity: Individually tailored Time: At least 30 min Type: Balance; Functional mobility	6 weeks	-	-	Balance: MiniBESTest; Number of falls Functional mobility: TUG; FTSTS; 10MWT; Walking speed	TUG, FTSTS, 10MWT scores, and Walking speed were significantly improved
26	Hidecker et al., 2022 United States	One group pre-post	15	Age: 74.1 (10.4) Disease duration: 9.3 (1.2) H&Y stage: 1.5 to 3	Synchronous	Zoom videoconference-guided exercise delivered via laptop computer	Frequency: 1 x / week Intensity: Individually tailored Time: 60 min Type: Endurance; Strength; Balance; Relaxation; Flexibility	8 weeks	-	-	Functional mobility: 30-sec STS Quality of life: PDQ-39 (Summary index and Mobility score)	30-sec STS scores were significantly improved
27	Holmes et al., 2013 Canada	One group pre-post	15	Age: 66.6 (6.1) Disease duration: 8.5 (3.9) H&Y stage: 1.5 to 2.5	Asynchronous	Nintendo Wii-guided unsupervised exercise (exergaming). Telephone monitoring was done weekly.	Frequency: 3 x / week Intensity: Individually tailored Time: 30 min Type: Balance	12 weeks		-	Balance: ABC	There was no improvement
28	Kwok et al., 2022 Hong Kong	One group pre-post	8	Age: 63.1 (5.4) Disease duration: Not reported H&Y stage: 3	Synchronous	Videoconference guided mindfulness group yoga	Frequency: Intensity: Time: Type: Relaxation	4 weeks	-	-	Balance: BBS; ABC Functional mobility: NFOGQ Quality of life: PDQ-8	There was significant improvement in BBS scores
29	Landers et al., 2020 United States	One group pre-post	47	Age: 62.1 (9.6) Disease duration: 3.3 (2.5) H&Y stage: Not mentioned	Asynchronous	9zet Parkinson’s Therapy mobile application-guided exercise delivered via smartphone	Frequency: 3 – 5 x / week Intensity: Individually tailored Time: 30 – 60 min Type: Endurance; Strength; Balance; Functional mobility; Flexibility; Relaxation	12 weeks	-	-	Functional mobility: 30-sec STS; TUG Quality of life: PDQ-8	The 30-sec STS, TUG, and PDQ-8 scores were significantly improved
30	Lavoie et al., 2021 Canada	One group pre-post	11	Age: 69.2 (3.6) Disease duration: Not mentioned H&Y stage: 2 to 3	Synchronous	Teraplus platform videoconference-guided exercise delivered via unspecified device. Caregiver supervised all sessions.	Frequency: 2 x / week Intensity: Individually tailored Time: 60 min Type: Functional mobility; Balance; Flexibility; Strength	8 weeks	3 months	-	Balance: MiniBESTest; FES-I Functional mobility: TUG; TUG-C; 6MWT Quality of life: PDQ-39	TUG-C and PDQ-39 scores were improved and retained at follow-up
31	Okusa et al., 2025 Japan	One group pre-post	56	Age: 73.8 (5.5) Disease duration: 6 (6.8) H&Y stage: 1 to 3	Synchronous	Zoom-guided prerecorded multimodal exercise	Frequency: 2x / week Intensity: Moderate Time: 40 min Type: Flexibility; Strength; Endurance; Functional mobility	6 months	-	-	Quality of life: PDQ-39 (Summary index and Mobility score)	PDQ-39 summary index scores were significantly improved
32	Potzulo et al., 2023 Italy	One group pre-post	20	Age: 66.9 (7.6) Disease duration: 7.4 (4) H&Y stage: 1 to 3	Asynchronous	Parkinson Rehab mobile application-guided exercise delivered via smartphone. Telephone monitoring was done twice a week.	Frequency: Daily Intensity: Individually tailored Time: 30 – 40 min Type: Functional mobility; Balance	8 weeks	1 month	-	Balance: MiniBESTest; FSST Functional mobility: SPPB; NFOGQ	MiniBESTest, FSST, and SPPB scores were significantly improved. Improvement s were maintained at follow-up.
33	Walton et al., 2022 Sweden	One group pre-post	23	Age: 70.4 (7.4) Disease duration: 9 (6.4) H&Y stage: Not reported	Synchronous	Zoom-guided Dance for PD program	Frequency: 1 x / week Intensity: Moderate Time: 60 min Type: Functional mobility	10 weeks	-	-	Quality of life: PDQ-39 (Summary index and Mobility score)	PDQ-39 summary index and mobility scores were significantly improved.
34	An et al., 2020 United States	Case series	2	Age: 75.5 (5) Disease duration: Not mentioned H&Y stage: 2 to 4	Asynchronous	Smarter Balance System mobile application-guided exercise delivered via smartphone mounted on a walker while wearing a custom belt which provides vibrotactile biofeedback	Frequency: 3 x / week Intensity: Individually tailored Time: 45 min Type: Balance	6 weeks	1 month	-	Balance: FES-I; ABC; DGI	There was no improvement in any outcomes
35	Carvalho et al., 2021 Canada	Case series	2	Age: 74.5 (0.5) Disease duration: 14.5 (2.5) H&Y stage: 3	Synchronous	Teraplus platform videoconference-guided Baduanjin Qigong exercise program via a touchscreen computer mounted on a tripod. Caregiver supervised all sessions.	Frequency: 2 x / week (supervised); 1 x / week (unsupervised) Intensity: Individually tailored Time: 30 – 60 min Type: Balance; Functional mobility; Flexibility; Relaxation	8 weeks	-	-	Balance: MiniBESTest Functional mobility: 10MWT; 2MWT; Gait speed Quality of life: PDQ-39 (Summary index and Mobility score)	MiniBESTest, Gait speed, and PDQ-39 summary index and mobility scores were clinically improved
36	Cornejo-Thumm et al., 2021 Israel	Case series	2	Age: 56.5 (10.5) Disease duration: 16 (1) H&Y stage: 3	Synchronous	Skype videoconference-guided VR treadmill training (exergaming) delivered via Microsoft Kinect and TV	Frequency: 1 x / week Intensity: Individually tailored Time: 30 – 75 min Type: Functional mobility	12 months	-	-	Balance: ABC Functional mobility: Gait speed	Gait speed and ABC scores were clinically improved
37	Chatto et al., 2018 United States	Case report	1	Age: 67 Disease duration: 2 H&Y stage: 2	Asynchronous	START system - DVD-guided LSVT-BIG program performed via Microsoft Kinect feedback delivered via touchscreen computer.	Frequency: Daily Intensity: Individually tailored Time: 30 min Type: Functional mobility; Balance	4 weeks	3 months	In-clinic balance training	Balance: ABC Functional mobility: ITUG	ABC scores were significantly improved in the therapist-supervised group.

*Note*. ABC – Activities-specific Balance Confidence Scale; ACSM – American College of Sports Medicine; AR – Augmented Reality; BBS – Berg Balance Scale; CG – Control Group; DGI – Dynamic Gait Index; DVD – Digital Video Disc; FES-I – Falls Efficacy Scale – International; FOG – Freezing of Gait; FRT – Functional Reach Test; FSST – Four Square Step Test; FTSTS – Five Times Sit to Stand; HR – Heart Rate; HRR – Heart Rate Reserve; IG – Intervention Group; ITUG – Instrumented Timed Up and Go; LSVT-BIG – Lee Silverman Voice Treatment – BIG; NFOGQ – New Freezing of Gait Questionnaire; PANAS – Positive and Negative Affect Schedule; PD – Parkinson’s Disease; PDQ-39 – Parkinson’s Disease Questionnaire – 39 items; PDQ-8 – Parkinson’s Disease Questionnaire – 8 items; PT – Physiotherapy; RPE – Rating of Perceived Exertion; SD – Standard Deviation; SF-12 – Short Form Health Survey – 12 items; SF-36 – Short Form Health Survey – 36 items; SPPB – Short Physical Performance Battery; START – System for Telerehabilitation and Remote Training; STS – Sit to Stand; TR – Telerehabilitation; TUG – Timed Up and Go; TUG-C – Timed Up and Go – Cognitive; TV – Television; VR – Virtual Reality; VRRS – Virtual Reality Rehabilitation System

## Data Availability

The data from the review are available from the corresponding author upon reasonable request.

## Appendix A Search Strategy

**Review Title**

Effect of home-based telerehabilitation on balance and functional mobility in Parkinson’s disease: a systematic review

**Review Questions**

1. Does home-based telerehabilitation have an effect on balance, functional mobility, and quality of life in persons with Parkinson’s disease?
2. What are the factors influencing the effect of home-based telerehabilitation on balance, functional mobility, and quality of life?

**Search Strategy**

**Table:**

	POPULATION	INTERVENTION	OUTCOMES	SETTING
**MeSH terms**	Parkinsonian Disorders	Telerehabilitation Remote Consultation Smartphone Cell phone Telephone Internet Telecommunications	Postural Balance Mobility Limitation Quality of Life Accidental Falls Movement Locomotion Gait Gait Disorders, Neurologic Walking Speed	Home Environment Home care services
**Key words**	Parkinson*	Telehealth Telephysiotherapy Tele physical therapy Remote physiotherapy Remote physical therapy Remote rehabilitation Virtual physiotherapy Virtual physical therapy Virtual rehabilitation e-Health mHealth Mobile Health Digital Health Videoconferenc* Teleconferenc* Video call* Video chat* Online Teleconsultation Mobile phone Remote* monitor* Remote* supervis*	Balance Postural instability Postural stability Postural control Postural equilibrium Body equilibrium Mobility Gait Speed Ambulation Fall* Fall rate Rate of fall* Fear of fall* Fall risk Risk of fall* Fall and slip Slip and fall Fall injur* Fall-related injur* Fall frequency Frequency of fall* Health-related Quality of Life	Home* Home-based

**PubMed Search String = 206**

Date: 31 May 2025

**Table:**

**P**	(“Parkinsonian Disorders”[MeSH Terms] OR Parkinson*[All Fields])
**I**	(Telerehabilitation[MeSH Terms] OR “Tele Rehabilitation”[All Fields] OR “Tele-Rehabilitation”[All Fields] OR Teleneurorehabilitation[All Fields] OR “Tele Neurorehabilitation”[All Fields] OR “Tele-Neurorehabilitation”[All Fields] OR “Remote Consultation”[MeSH Terms] OR Teleconsultation[All Fields] OR “Tele Consultation”[All Fields] OR “Tele-Consultation”[All Fields] OR Telehealth[All Fields] OR “Tele Health”[All Fields] OR “Tele-Health”[All Fields] OR “Remote Health”[All Fields] OR Telephysiotherapy[All Fields] OR “Tele-Physiotherapy”[All Fields] OR “Tele Physical Therapy”[All Fields] OR “Remote Physiotherapy”[All Fields] OR “Remote Physical Therapy”[All Fields] OR “Remote Rehabilitation”[All Fields] OR “Virtual Physiotherapy”[All Fields] OR “Virtual Physical Therapy”[All Fields] OR “Virtual Rehabilitation”[All Fields] OR eHealth[All Fields] OR mHealth[All Fields] OR “Mobile Health”[All Fields] OR “Digital Health”[All Fields] OR Videoconferenc*[All Fields] OR “Video-Conferenc*”[All Fields] OR “Video Conferenc*”[All Fields] OR Teleconferenc*[All Fields] OR “Tele-Conferenc*”[All Fields] OR “Tele Conferenc*”[All Fields] OR “Video Call*”[All Fields] OR “Video-Call*”[All Fields] OR “Video Chat*”[All Fields] OR “Video-Chat*”[All Fields] OR “Remote* Supervis*”[All Fields] OR “Remote* Monitor*”[All Fields] OR Online[All Fields] OR Internet[MeSH Terms] OR Telecommunications[MeSH Terms] OR Smartphone[MeSH Terms] OR “Smart Phone”[All Fields] OR “Cell Phone”[MeSH Terms] OR Telephone[MeSH Terms] OR “Smart Phone”[All Fields] OR “Mobile Phone”[All Fields])
**O**	(“Postural Balance”[MeSH Terms] OR “Mobility Limitation”[MeSH Terms] OR “Quality of Life”[MeSH Terms] OR “Accidental Falls”[MeSH Terms] OR Movement[MeSH Terms] OR Locomotion[MeSH Terms] OR Gait[MeSH Terms] OR “Gait Disorders, Neurologic”[MeSH Terms] OR “Walking Speed”[MeSH Terms] OR Balance[All Fields] OR Mobility[All Fields] OR “Functional Mobility”[All Fields] OR “Postural Instability”[All Fields] OR “Postural Stability”[All Fields] OR “Postural Control”[All Fields] OR “Postural Equilibrium”[All Fields] OR “Body Equilibrium”[All Fields] OR Fall*[All Fields] OR “Gait Speed”[All Fields] OR Ambulation[All Fields]OR “Fall Rate”[All Fields] OR “Rate of Fall*”[All Fields] OR “Fear of Fall*”[All Fields] OR “Fall Risk”[All Fields] OR “Risk of Fall*”[All Fields] OR “Fall and Slip”[All Fields] OR “Slip and Fall”[All Fields] OR “Fall Injur*”[All Fields] OR “Fall-related Injur*”[All Fields] OR “Fall Frequency”[All Fields] OR “Frequency of Fall*”[All Fields] OR “Health Related Quality of Life”[All Fields] OR “Health-Related Quality of Life”[All Fields] OR HRQOL[All Fields] OR QOL[All Fields])
**S**	“Home Environment”[MeSH Terms] OR “Home Care Services”[MeSH Terms] OR Home*[All Fields]
	((((“Parkinsonian Disorders”[MeSH Terms] OR Parkinson*[All Fields])) AND ((Telerehabilitation[MeSH Terms] OR “Tele Rehabilitation”[All Fields] OR “Tele-Rehabilitation”[All Fields] OR Teleneurorehabilitation[All Fields] OR “Tele Neurorehabilitation”[All Fields] OR “Tele-Neurorehabilitation”[All Fields] OR “Remote Consultation”[MeSH Terms] OR Teleconsultation[All Fields] OR “Tele Consultation”[All Fields] OR “Tele-Consultation”[All Fields] OR Telehealth[All Fields] OR “Tele Health”[All Fields] OR “Tele-Health”[All Fields] OR “Remote Health”[All Fields] OR Telephysiotherapy[All Fields] OR “Tele-Physiotherapy”[All Fields] OR “Tele Physical Therapy”[All Fields] OR “Remote Physiotherapy”[All Fields] OR “Remote Physical Therapy”[All Fields] OR “Remote Rehabilitation”[All Fields] OR “Virtual Physiotherapy”[All Fields] OR “Virtual Physical Therapy”[All Fields] OR “Virtual Rehabilitation”[All Fields] OR eHealth[All Fields] OR mHealth[All Fields] OR “Mobile Health”[All Fields] OR “Digital Health”[All Fields] OR Videoconferenc*[All Fields] OR “Video-Conferenc*”[All Fields] OR “Video Conferenc*”[All Fields] OR Teleconferenc*[All Fields] OR “Tele-Conferenc*”[All Fields] OR “Tele Conferenc*”[All Fields] OR “Video Call*”[All Fields] OR “Video-Call*”[All Fields] OR “Video Chat*”[All Fields] OR “Video-Chat*”[All Fields] OR “Remote* Supervis*”[All Fields] OR “Remote* Monitor*”[All Fields] OR Online[All Fields] OR Internet[MeSH Terms] OR Telecommunications[MeSH Terms] OR Smartphone[MeSH Terms] OR “Smart Phone”[All Fields] OR “Cell Phone”[MeSH Terms] OR Telephone[MeSH Terms] OR “Smart Phone”[All Fields] OR “Mobile Phone”[All Fields]))) AND ((“Postural Balance”[MeSH Terms] OR “Mobility Limitation”[MeSH Terms] OR “Quality of Life”[MeSH Terms] OR “Accidental Falls”[MeSH Terms] OR Movement[MeSH Terms] OR Locomotion[MeSH Terms] OR Gait[MeSH Terms] OR “Gait Disorders, Neurologic”[MeSH Terms] OR “Walking Speed”[MeSH Terms] OR Balance[All Fields] OR Mobility[All Fields] OR “Functional Mobility”[All Fields] OR “Postural Instability”[All Fields] OR “Postural Stability”[All Fields] OR “Postural Control”[All Fields] OR “Postural Equilibrium”[All Fields] OR “Body Equilibrium”[All Fields] OR Fall*[All Fields] OR “Gait Speed”[All Fields] OR Ambulation[All Fields]OR “Fall Rate”[All Fields] OR “Rate of Fall*”[All Fields] OR “Fear of Fall*”[All Fields] OR “Fall Risk”[All Fields] OR “Risk of Fall*”[All Fields] OR “Fall and Slip”[All Fields] OR “Slip and Fall”[All Fields] OR “Fall Injur*”[All Fields] OR “Fall-related Injur*”[All Fields] OR “Fall Frequency”[All Fields] OR “Frequency of Fall*”[All Fields] OR “Health Related Quality of Life”[All Fields] OR “Health-Related Quality of Life”[All Fields] OR HRQOL[All Fields] OR QOL[All Fields]))) AND (“Home Environment”[MeSH Terms] OR “Home Care Services”[MeSH Terms] OR Home*[All Fields])

**CINAHL Search String = 1,719**

Date: 31 May 2025

**Table:**

**P**	(MH “Parkinsonian Disorders”) OR (TX “Parkinson*”)
**I**	(MH “Telerehabilitation”) OR (MH “Telehealth”) OR (MH “Telemedicine”) OR (MH “Internet”) OR (MH “Internet-Based Intervention”) OR (MH “Telecommunications”) OR (MH “Cellular Phone”) OR (MH “Telephone”) OR (MH “Smartphone”) OR (MH “Videoconferencing”) OR (MH “Teleconferencing”) OR (MH “Remote Consultation”) OR (TX “Tele-Rehabilitation”) OR (TX “Tele Rehabilitation”) OR (TX “Teleneurorehabilitation”) OR (TX “Tele Neurorehabilitation”) OR (TX “Tele-Neurorehabilitation”) OR (TX “Teleconsultation”) OR (TX “Tele-Consultation”) OR (TX “Tele Consultation”) OR (TX “Tele-Health”) OR (TX “Tele Health”) OR(TX “Remote Health”) OR (TX “Telephysiotherapy”) OR (TX “Tele physiotherapy”) OR (TX “Tele-physiotherapy”) OR (TX “Tele physical therapy”) OR (TX “Remote physiotherapy”) OR (TX “Remote Physical Therapy”) OR (TX “Remote Rehabilitation”) OR (TX “Virtual Physiotherapy”) OR (TX “Virtual Physical Therapy”) OR (TX “Virtual Rehabilitation”) OR (TX “eHealth”) OR (TX “mHealth”) OR (TX “Mobile Health”) OR (TX “Digital Health”) OR (TX “Videoconferenc*”) OR (TX “Video Conferenc*”) OR (TX “Video-Conferenc*”) OR (TX “Teleconferenc*”) OR (TX “Tele Conferenc*”) OR (TX “Tele-Conferenc*”) OR (TX “Video Call*”) OR (TX “Video-call*”) OR (TX “Video Chat*”) OR (TX “Video-Chat*”) OR (TX “Remote* Supervis*”) OR (TX “Remote* Monitor*”)
**O**	(MH “Balance, Postural”) OR (MH “Balance Training, Physical”) OR (MH “Physical Mobility”) OR (MH “Gait”) OR (MH “Gait Disorders, Neurologic”) OR (MH “Gait Training”) OR (MH “Walking Speed”) OR (MH “Quality of Life”) OR (MH “Accidental Falls”) OR (TX “Balance”) OR (TX “Postural Instability”) OR (TX “Postural Stability”) OR (TX “Postural Control”) OR (TX “Postural Equilibrium”) OR (TX “Body Equilibrium”) OR (TX “Mobility”) OR (TX “Functional Mobility”) OR (TX “Gait Speed”) OR (TX “Ambulation”) OR (TX “Locomotion”) OR (TX “Fall*”) OR (TX “Fall Rate”) OR (TX “Rate of Fall*”) OR (TX “Fear of Fall”) OR (TX “Fall Risk”) OR (TX “Risk of Fall*”) OR (TX “Fall and Slip”) OR (TX “Slip and Fall”) OR (TX “Fall Injur*”) OR (TX “Fall-Related Injur*”) OR (TX “Fall Frequency”) OR (TX “Frequency of Fall*”) OR (TX “Health-Related Quality of Life”) OR (TX “ Health Related Quality of Life “)
**S**	(MH “Home Rehabilitation”) OR (MH “Home Health Care”) OR (MH “Home Environment”) OR (MH “Home Physical Therapy”) OR (TX “Home*”)
	( (MH “Parkinsonian Disorders”) OR (TX “Parkinson*”) ) AND ( (MH “Telerehabilitation”) OR (MH “Telehealth”) OR (MH “Telemedicine”) OR (MH “Internet”) OR (MH “Internet-Based Intervention”) OR (MH “Telecommunications”) OR (MH “Cellular Phone”) OR (MH “Telephone”) OR (MH “Smartphone”) OR (MH “Videoconferencing”) OR (MH “Teleconferencing”) OR (MH “Remote Consultation”) OR (TX “Tele-Rehabilitation”) OR (TX “Tele Rehabilitation”) OR (TX “Teleneurorehabilitation”) OR (TX “Tele Neurorehabilitation”) OR (TX “Tele-Neurorehabilitation”) OR (TX “Teleconsultation”) OR (TX “Tele-Consultation”) OR (TX “Tele Consultation”) OR (TX “Tele-Health”) OR (TX “Tele Health”) OR(TX “Remote Health”) OR (TX “Telephysiotherapy”) OR (TX “Tele physiotherapy”) OR (TX “Tele-physiotherapy”) OR (TX “Tele physical therapy”) OR (TX “Remote physiotherapy”) OR (TX “Remote Physical Therapy”) OR (TX “Remote Rehabilitation”) OR (TX “Virtual Physiotherapy”) OR (TX “Virtual Physical Therapy”) OR (TX “Virtual Rehabilitation”) OR (TX “eHealth”) OR (TX “mHealth”) OR (TX “Mobile Health”) OR (TX “Digital Health”) OR (TX “Videoconferenc*”) OR (TX “Video Conferenc*”) OR (TX “Video-Conferenc*”) OR (TX “Teleconferenc*”) OR (TX “Tele Conferenc*”) OR (TX “Tele-Conferenc*”) OR (TX “Video Call*”) OR (TX “Video-call*”) OR (TX “Video Chat*”) OR (TX “Video-Chat*”) OR (TX “Remote* Supervis*”) OR (TX “Remote* Monitor*”) ) AND ( (MH “Balance, Postural”) OR (MH “Balance Training, Physical”) OR (MH “Physical Mobility”) OR (MH “Gait”) OR (MH “Gait Disorders, Neurologic”) OR (MH “Gait Training”) OR (MH “Walking Speed”) OR (MH “Quality of Life”) OR (MH “Accidental Falls”) OR (TX “Balance”) OR (TX “Postural Instability”) OR (TX “Postural Stability”) OR (TX “Postural Control”) OR (TX “Postural Equilibrium”) OR (TX “Body Equilibrium”) OR (TX “Mobility”) OR (TX “Functional Mobility”) OR (TX “Gait Speed”) OR (TX “Ambulation”) OR (TX “Locomotion”) OR (TX “Fall*”) OR (TX “Fall Rate”) OR (TX “Rate of Fall*”) OR (TX “Fear of Fall”) OR (TX “Fall Risk”) OR (TX “Risk of Fall*”) OR (TX “Fall and Slip”) OR (TX “Slip and Fall”) OR (TX “Fall Injur*”) OR (TX “Fall-Related Injur*”) OR (TX “Fall Frequency”) OR (TX “Frequency of Fall*”) OR (TX “Health-Related Quality of Life”) OR (TX “ Health Related Quality of Life “) ) AND ( (MH “Home Rehabilitation”) OR (MH “Home Health Care”) OR (MH “Home Environment”) OR (MH “Home Physical Therapy”) OR (TX “Home*”) )

**Embase Search String = 406**

Date: 31 May 2025

**Table:**

**P**	‘parkinson disease’/exp OR ‘parkinson disease’ OR ‘parkinsonism’/exp OR ‘parkinsonism’ OR parkinson*
**I**	(‘telerehabilitation’/exp OR ‘telerehabilitation’ OR ‘telehealth’/exp OR ‘telehealth’ OR ‘telemedicine’/exp OR ‘telemedicine’ OR ‘teleconsultation’/exp OR ‘teleconsultation’ OR ‘internet’/exp OR ‘internet’ OR ‘web-based intervention’/exp OR ‘web-based intervention’) AND (‘telecommunication’/exp OR ‘telecommunication’) OR ‘internet’/exp OR ‘internet’ OR ‘online’/exp OR ‘online’ OR ‘smartphone’/exp OR ‘smartphone’ OR ‘mobile phone’/exp OR ‘mobile phone’ OR ‘telephone’/exp OR ‘telephone’ OR ‘videoconferencing’/exp OR ‘videoconferencing’ OR ‘teleconference’/exp OR ‘teleconference’ OR ‘telemonitoring’/exp OR ‘telemonitoring’ OR ‘mhealth’/exp OR ‘mhealth’ OR ‘mobile health application’/exp OR ‘mobile health application’ OR ‘digital health’/exp OR ‘digital health’ OR ‘digital health intervention’/exp OR ‘digital health intervention’ OR ‘video consultation’/exp OR ‘video consultation’
**O**	‘balance’/exp OR ‘balance’ OR ‘body equilibrium’/exp OR ‘body equilibrium’ OR ‘balance training’/exp OR ‘balance training’ OR ‘mobility’/exp OR ‘mobility’ OR ‘walking difficulty’/exp OR ‘walking difficulty’ OR ‘functional mobility’/exp OR ‘functional mobility’ OR ‘gait’/exp OR ‘gait’ OR ‘neurologic gait disorder’/exp OR ‘neurologic gait disorder’ OR ‘quality of life’/exp OR ‘quality of life’ OR ‘falling’/exp OR ‘falling’ OR ‘fall risk’/exp OR ‘fall risk’ OR ‘postural control’/exp OR ‘postural control’ OR ‘postural stability’/exp OR ‘postural stability’ OR ‘postural instability’/exp OR ‘postural instability’ OR ‘postural instability gait difficulty’/exp OR ‘postural instability gait difficulty’ OR ‘postural instability gait disorder’/exp OR ‘postural instability gait disorder’
**S**	‘home’/exp OR ‘home’ OR ‘home rehabilitation’/exp OR ‘home rehabilitation’ OR ‘home based exercise program’/exp OR ‘home based exercise program’ OR ‘home based exercise’/exp OR ‘home based exercise’ OR ‘home care’/exp OR ‘home care’ OR ‘home environment’/exp OR ‘home environment’ OR home*
	(‘parkinson disease’/exp OR ‘parkinson disease’ OR ‘parkinsonism’/exp OR ‘parkinsonism’ OR parkinson*) AND ((‘telerehabilitation’/exp OR ‘telerehabilitation’ OR ‘telehealth’/exp OR ‘telehealth’ OR ‘telemedicine’/exp OR ‘telemedicine’ OR ‘teleconsultation’/exp OR ‘teleconsultation’ OR ‘internet’/exp OR ‘internet’ OR ‘web-based intervention’/exp OR ‘web-based intervention’) AND (‘telecommunication’/exp OR ‘telecommunication’) OR ‘internet’/exp OR ‘internet’ OR ‘online’/exp OR ‘online’ OR ‘smartphone’/exp OR ‘smartphone’ OR ‘mobile phone’/exp OR ‘mobile phone’ OR ‘telephone’/exp OR ‘telephone’ OR ‘videoconferencing’/exp OR ‘videoconferencing’ OR ‘teleconference’/exp OR ‘teleconference’ OR ‘telemonitoring’/exp OR ‘telemonitoring’ OR ‘mhealth’/exp OR ‘mhealth’ OR ‘mobile health application’/exp OR ‘mobile health application’ OR ‘digital health’/exp OR ‘digital health’ OR ‘digital health intervention’/exp OR ‘digital health intervention’ OR ‘video consultation’/exp OR ‘video consultation’) AND (‘balance’/exp OR ‘balance’ OR ‘body equilibrium’/exp OR ‘body equilibrium’ OR ‘balance training’/exp OR ‘balance training’ OR ‘mobility’/exp OR ‘mobility’ OR ‘walking difficulty’/exp OR ‘walking difficulty’ OR ‘functional mobility’/exp OR ‘functional mobility’ OR ‘gait’/exp OR ‘gait’ OR ‘neurologic gait disorder’/exp OR ‘neurologic gait disorder’ OR ‘quality of life’/exp OR ‘quality of life’ OR ‘falling’/exp OR ‘falling’ OR ‘fall risk’/exp OR ‘fall risk’ OR ‘postural control’/exp OR ‘postural control’ OR ‘postural stability’/exp OR ‘postural stability’ OR ‘postural instability’/exp OR ‘postural instability’ OR ‘postural instability gait difficulty’/exp OR ‘postural instability gait difficulty’ OR ‘postural instability gait disorder’/exp OR ‘postural instability gait disorder’) AND (‘home’/exp OR ‘home’ OR ‘home rehabilitation’/exp OR ‘home rehabilitation’ OR ‘home based exercise program’/exp OR ‘home based exercise program’ OR ‘home based exercise’/exp OR ‘home based exercise’ OR ‘home care’/exp OR ‘home care’ OR ‘home environment’/exp OR ‘home environment’ OR home*)

**Scopus Search String = 1517**

Date: 31 May 2025

**Table:**

**P**	TITLE-ABS-KEY ( Parkinson* )
**I**	ALL ( telehealth OR “Tele health” OR telerehabilitation OR “Tele rehabilitation” OR telemedicine OR “Tele medicine” OR “Telephysiotherapy” OR “Tele physiotherapy” OR “Tele physical therapy” OR teleconsultation OR “Tele consultation” OR “Remote Rehabilitation” OR “Remote Consultation” OR “Remote Physiotherapy” OR “Remote Physical Therapy” OR “Remote* Supervis*” OR “Remote* Monitor*” OR teleconferenc* OR “Tele conferenc*” OR “Videoconference*” OR “Video conference*” OR “Virtual Rehabilitation” OR “Virtual Physiotherapy” OR “Virtual Physical Therapy” OR “eHealth” OR “mHealth” OR “Mobile Health” OR “Digital Health” OR “Remote Health” )
**O**	ALL ( Balance OR “Postural Control” OR “Postural Instability” OR “Postural Stability” OR Fall* OR Mobility OR Gait OR Ambulation OR “Quality of Life” )
**S**	ALL ( Home* )
	TITLE-ABS-KEY ( parkinson* ) AND ALL ( telehealth OR “Tele health” OR telerehabilitation OR “Tele rehabilitation” OR telemedicine OR “Tele medicine” OR “Telephysiotherapy” OR “Tele physiotherapy” OR “Tele physical therapy” OR teleconsultation OR “Tele consultation” OR “Remote Rehabilitation” OR “Remote Consultation” OR “Remote Physiotherapy” OR “Remote Physical Therapy” OR “Remote* Supervis*” OR “Remote* Monitor*” OR teleconferenc* OR “Tele conferenc*” OR “Videoconference*” OR “Video conference*” OR “Virtual Rehabilitation” OR “Virtual Physiotherapy” OR “Virtual Physical Therapy” OR “eHealth” OR “mHealth” OR “Mobile Health” OR “Digital Health” OR “Remote Health” ) AND ALL ( balance OR “Postural Control” OR “Postural Instability” OR “Postural Stability” OR fall* OR mobility OR gait OR ambulation OR “Quality of Life” ) AND ALL ( home* )

**Web of Science Search String = 168**

Date: 22 May 2025

**Table:**

**P**	ALL=(Parkinson*)
**I**	ALL=(Telerehabilitation OR “Tele Rehabilitation” OR “Tele-Rehabilitation” OR Telehealth OR “Tele-Health” OR “Tele Health” OR “Telemedicine” OR “Tele-Medicine” OR “Tele Medicine” OR “Teleconsultation” OR “Tele Consultation” OR “Tele-Consultation” OR Telephysiotherapy OR “Tele-physiotherapy” OR “Tele Physiotherapy” OR “Tele physical therapy” OR “Remote Consultation” OR eHealth OR mHealth OR “Digital Health” OR “Remote Health” OR “Videoconferenc*” OR “Teleconferenc*” OR “Video Conferenc*” OR “Tele Conferenc*” OR “Video-Conferenc*” OR “Tele-Conferenc*” OR “Remote* Supervis*” OR “Remote* Monitor*” OR “Remote Rehabilitation” OR “Virtual Rehabilitation” OR “Virtual Physiotherapy” OR “Virtual Physical Therapy”)
**O**	ALL=(Balance OR “Postural Control” OR “Postural Instability” OR “Postural Stability” OR Mobility OR Gait OR Walking OR Ambulation OR Fall* OR “Quality of Life”)
**S**	ALL=(Home*)
	(ALL=(Parkinson*)) AND (ALL=(Telerehabilitation OR “Tele Rehabilitation” OR “Tele-Rehabilitation” OR Telehealth OR “Tele-Health” OR “Tele Health” OR “Telemedicine” OR “Tele-Medicine” OR “Tele Medicine” OR “Teleconsultation” OR “Tele Consultation” OR “Tele-Consultation” OR Telephysiotherapy OR “Tele-physiotherapy” OR “Tele Physiotherapy” OR “Tele physical therapy” OR “Remote Consultation” OR Teleconsultation OR “Tele consultation” OR eHealth OR mHealth OR “Digital Health” OR “Remote Health” OR “Videoconferenc*” OR “Teleconferenc*” OR “Video Conferenc*” OR “Tele Conferenc*” OR “Video-Conferenc*” OR “Tele-Conferenc*” OR “Remote* Supervis*” OR “Remote* Monitor*” OR “Remote Rehabilitation” OR “Virtual Rehabilitation” OR “Virtual Physiotherapy” OR “Virtual Physical Therapy”)) AND (ALL=(Balance OR “Postural Control” OR “Postural Instability” OR “Postural Stability” OR Mobility OR Gait OR Walking OR Ambulation OR Fall* OR “Quality of Life”)) AND (ALL=(Home*))

**Cochrane CENTRAL = 546**

Date: 31 May 2025

**Table:**

**P**	Parkinson* in Title Abstract Keyword
**I**	Telerehabilitation OR Telehealth OR Telemedicine OR eHealth OR mHealth OR Teleconferenc* OR Videoconferenc* OR Telephysiotherapy OR “Tele physical therapy” OR “Remote Physiotherapy” OR “Remote Physical Therapy” OR “Remote Rehabilitation” in All Text
**O**	Balance OR Mobility OR Gait OR Fall* OR “Quality of Life” in All Text
**S**	Home in All Text
	Parkinson* in Title Abstract Keyword AND Telerehabilitation OR Telehealth OR Telemedicine OR eHealth OR mHealth OR Teleconferenc* OR Videoconferenc* OR Telephysiotherapy OR “Tele physical therapy” OR “Remote Physiotherapy” OR “Remote Physical Therapy” OR “Remote Rehabilitation” in All Text AND Balance OR Mobility OR Gait OR Fall* OR “Quality of Life” in All Text AND Home in All Text

**OvidSP = 840**

Date: 31 May 2025

**Table:**

**P**	Parkinson*.mp. [mp=title, abstract, full text, caption text]
**I**	(Telerehabilitation or Telehealth or Telemedicine or Teleconsultation or Telephysiotherapy or “Tele physical therapy” or “Remote Consultation” or eHealth or mHealth or “Digital Health” or “Mobile Health” or “Remote Health” or “Videoconferenc*” or “Teleconferenc*”).mp. [mp=title, abstract, full text, caption text]
**O**	(Balance or “Postural Balance” or “Postural Instability” or “Postural Stability” or “Functional Mobility” or Mobility or Gait or Walking or “Accidental Falls” or Fall* or “Quality of Life”).mp. [mp=title, abstract, full text, caption text]
**S**	Home*.mp. [mp=title, abstract, full text, caption text]

**ProQuest Search String = 1,632**

Date: 31 May 2025

**Table:**

**P**	Exact (“Parkinsonian Disorders”) OR Parkinson*
**I**	Exact(“Telemedicine” OR “Telephone” OR “Telecommunications” OR “Remote Consultation”) OR (Telerehabilitation OR “Tele Rehabilitation” OR “Tele-Rehabilitation” OR Telehealth OR “Tele Health” OR “Tele-Health” OR “Tele medicine” OR “Telephysiotherapy” OR “Tele physiotherapy” OR “Tele physical therapy” OR Teleconsultation OR “Tele consultation” OR “Remote Rehabilitation” OR “Remote Consultation” OR “Remote Physiotherapy” OR “Remote Physical Therapy” OR “Remote* Supervis*” OR “Remote* Monitor*” OR Teleconferenc* OR “Tele conferenc*” OR “Videoconference*” OR “Video conference*” OR “Virtual Rehabilitation” OR “Virtual Physiotherapy” OR “Virtual Physical Therapy” OR “eHealth” OR “mHealth” OR “Mobile Health” OR “Digital Health” OR “Remote Health”)
**O**	Exact (“Locomotion” OR “Mobility Limitation” OR “Movement” OR “Gait” OR “Quality of Life” OR “Gait Disorders, Neurologic” OR “Postural Balance” OR “Accidental Falls”) OR (Mobility OR Fall*)
**S**	Exact (“Home Care Services”) OR Home*
	(Exact(“Parkinsonian Disorders”) OR Parkinson*) AND (Exact(“Telemedicine” OR “Remote Consultation”) OR (Telerehabilitation OR “Tele Rehabilitation” OR “Tele-Rehabilitation” OR Telehealth OR “Tele Health” OR “Tele-Health” OR “Tele medicine” OR “Telephysiotherapy” OR “Tele physiotherapy” OR “Tele physical therapy” OR Teleconsultation OR “Tele consultation” OR “Remote Rehabilitation” OR “Remote Consultation” OR “Remote Physiotherapy” OR “Remote Physical Therapy” OR “Remote* Supervis*” OR “Remote* Monitor*” OR Teleconferenc* OR “Tele conferenc*” OR “Videoconference*” OR “Video conference*” OR “Virtual Rehabilitation” OR “Virtual Physiotherapy” OR “Virtual Physical Therapy” OR “eHealth” OR “mHealth” OR “Mobile Health” OR “Digital Health” OR “Remote Health”)) AND (Exact(“Locomotion” OR “Mobility Limitation” OR “Movement” OR “Gait” OR “Quality of Life” OR “Gait Disorders, Neurologic” OR “Postural Balance” OR “Accidental Falls”) OR (Mobility OR Fall*)) AND (Exact(“Home Care Services”) OR Home*)

**PEDro Search String = 26**

Date: 22 May 2025

**Table:**

**P**	Parkinson*
**I**	Tele*
**O**	
**S**	

## Appendix B Systematic Review Quality Assessment

**Table:**

Sr No	Study	Randomization Process	Deviations from Intended Interventions	Missing Outcome Data	Measurement of the Outcome	Selection of the Reported Result	Overall Risk of Bias
1	Atterbury et al	High	Low	High	Some concerns	Low	High
2	Dhamija et al	Low	Low	Low	Low	Low	Low
3	Ellis et al	Low	Low	Low	Low	Low	Low
4	Flynn et al	Low	Low	Some concerns	Some concerns	Some concerns	Some concerns
5	Gandolfi et al	Low	Low	Low	Low	Low	Low
6	Ge et al	Some concerns	Low	Low	Low	Low	Some concerns
7	Ginis et al	Some concerns	Low	Low	Some concerns	Low	Some concerns
8	Goffredo et al	Low	Low	Some concerns	Low	Low	Some concerns
9	Gondim et al	Some concerns	Low	Some concerns	Some concerns	Low	Some concerns
10	Johnson et al	Low	Low	Some concerns	Some concerns	Low	Some concerns
11	Khalil et al	Some concerns	Low	Low	Low	Low	Some concerns
12	Ramos et al	Low	Low	Some concerns	Some concerns	Some concerns	Some concerns
13	van der Kolk et al	Low	Low	Low	Low	Low	Low
14	Vasconcellos et al	Low	Low	Some concerns	Some concerns	Low	Some concerns
